# Deep learning-based multi-class misbehavior classification in fog-enabled vehicular ad hoc networks

**DOI:** 10.1038/s41598-026-66695-8

**Published:** 2026-09-05

**Authors:** Hadeer Kamal, Amira Y. Haikal, Mahmoud M. Saafan

**Affiliations:** 1 Computers and Control Systems Engineering Department, Faculty of Engineering, Mansoura University, Mansoura, Egypt; 2 Faculty of Engineering, Mansoura National University, Mansoura, Egypt

**Keywords:** Fog computing, VANET, Data dissemination, Delay-sensitive, Deep learning, Engineering, Mathematics and computing

## Abstract

Traffic collisions and congestion represent significant challenges within intelligent transportation systems (ITS). Consequently, a vehicular ad-hoc network (VANET) has been established. Numerous architectures have been incorporated into VANETs to manage the extensive data generated by vehicles. Collaboration with fog computing is vital, particularly for applications requiring real-time processing. In addition, there is a growing demand for advanced intrusion detection methods. These techniques are employed to identify the optimal response that the fog server should provide based on data received from a vehicle. As the network continues to grow, the amount of data needing analysis also increases. Thus, deep learning approaches become increasingly efficient. The requirement for feature selection is reduced when leveraging deep learning techniques. This work uses two deep learning-based misbehavior classification schemes for intrusion detection in VANETs: Long Short-Term Memory (LSTM) and Convolutional Neural Networks (CNN). The vehicular data that the Roadside Units (RSUs) obtain is initially sent to the fog server for preprocessing and classification. This study suggests four classification methods, which include 4 LSTM, 2 LSTM-2 CNN, 3 CNN-1 LSTM, and 2 CNN-1 LSTM within two main classifiers. The first classifier uses them to classify each type of vehicle, while the second uses them to classify generic behavior categories. The results of the first classifier were higher than those of the existing work. The precision ranges from 94% to 98%. The recall ranges from 92% to 97%. The F1 scores range from 94% to 97%. The second classifier also scores the best results compared to other works. The precision, recall, and F1-score range from 99% to 99.66%. The time and space complexity are calculated for each model in each classifier.

## Introduction

Recent theoretical advancements have proven that the Internet of Things is a contemporary reality. Establishing a transportation system that is secure, dependable, and safe is vital, especially in smart cities. The primary problem is mitigating accidents. Despite all governmental regulations and statutes, the probability of traffic accidents has escalated^1^. Recently, the automotive industry has seen significant transformations. The sixth generation (6G) is expected to play a crucial role in enabling connected autonomous vehicles in the future.

Vehicles are equipped with communication devices to interact with each other. Therefore, they take proper action based on the messages received. Consequently, vehicular ad-hoc networks (VANETs), a subset of mobile ad-hoc networks (MANETs), are set up to facilitate communication among vehicles. They introduce chances for improving road safety^2^.

**Fig. 1:**
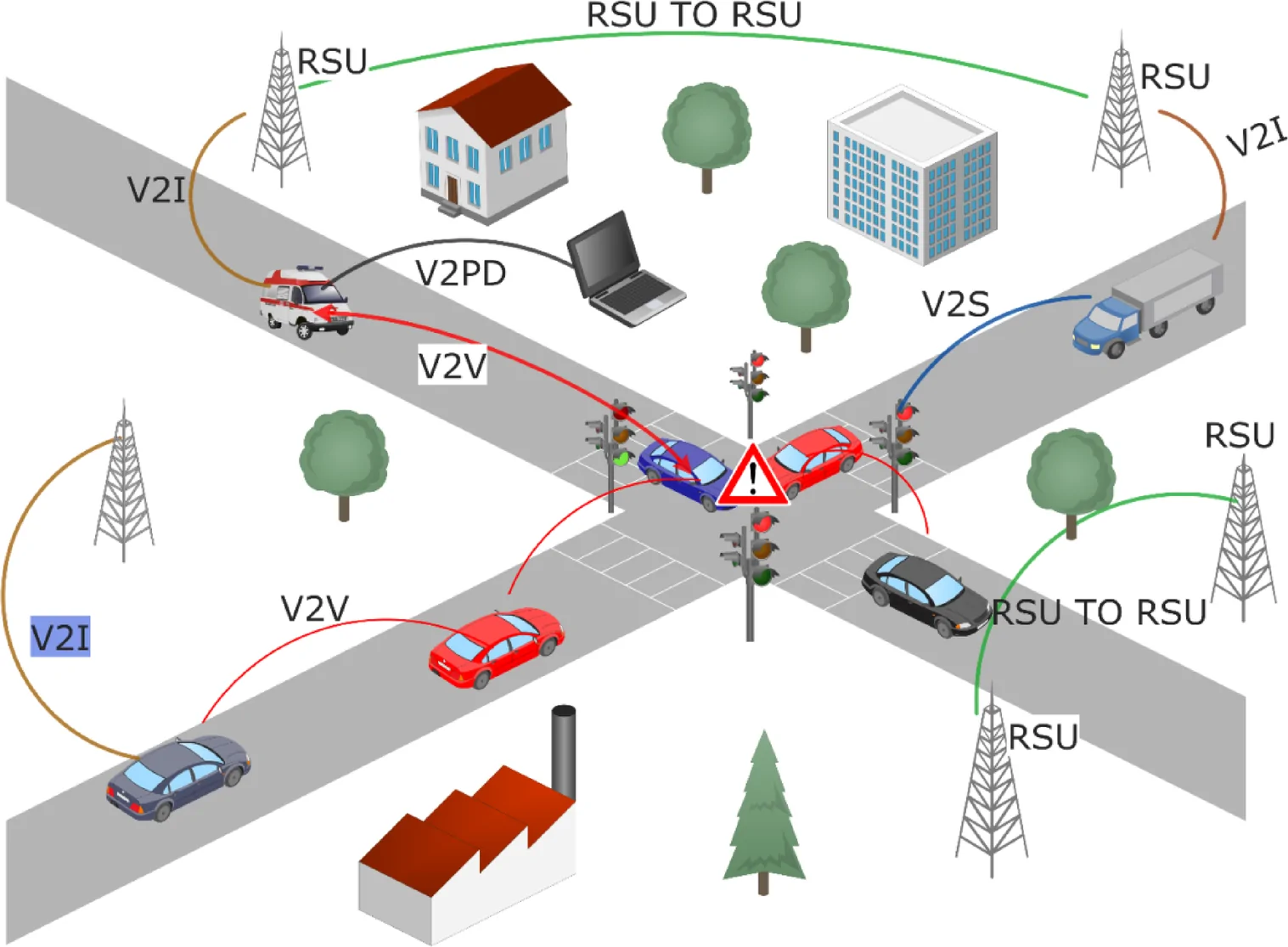
Overview of various communication types in VANETs.

VANETs are decentralized, infrastructure-less ad-hoc networks. They are used for V2X communication. They consist of a set of mobile (vehicles) and fixed (roadside units) entities working together to exchange vital information about road conditions and other vehicles^1,3^. Figure 1 presents an abstract representation of the architecture of VANETs. It also shows the different communications between objects in VANETs. Data dissemination in VANET is conducted via vehicle-to-vehicle (V2V), vehicle-to-infrastructure (V2I), infrastructure-to-vehicle (I2V) communications, vehicle-to-sensor (V2S), and vehicle-to-personal device (V2PD).

Messages are used to send data. There are two types of messages:

- PERIODIC BEACON MESSAGES: Basic safety messages (BSM) beacons are used to check if devices are connected. They guarantee that a certain car is within the range of a server or an RSU. Beacon communications provide the vehicle’s direction, speed, and current position.
- Event-Driven Messages: Event-driven safety messages are generated at once as an event occurs. These messages can be query-start, data collecting, emergency reporting, and accident notifications. Messages like these can be sent to RSUs through V2I or V2V communication^4^.

As shown in Fig. 1, vehicles can join or leave the group without generating any pre-indication. Additionally, modern vehicles are equipped with hundreds of sensors that generate massive amounts of data. The number of vehicles in the United States alone is above 250 million. Each car generates 20 GB of data every hour^5^. This presents various challenges in terms of data gathering, analysis, aggregation, processing, and storage. The data must be properly analyzed for various innovative and smart traffic management applications. As a result, the necessity to integrate VANETs with other architectures has arisen.

Combining cloud computing with VANETs can be an excellent approach to addressing this kind of issue. For a VANET system to function in cloud computing, it should be equipped with capabilities like location tracking, speed, mobility support, and rapid data transfer^6^. This integration is a blessing for many applications. Cloud computing helps vehicular communications in terms of computation and big data processing. However, high end-to-end latency limits the use of cloud computing for delay-sensitive tasks in VANETs. So, the architecture needs to be modified.

Cisco proposed fog computing, an extension of cloud computing, in 2011, to tackle the challenges previously outlined. Fog computing offers an additional layer of “infrastructure” between the edge devices. Devices with limited storage capacity, processing, and battery resources are utilized at the network edge of fog-based VANET^7^. Hence, the edge layer enables immediate engagement for applications that require low latency, such as emergency services. Also, it offloads massive traffic flows from the core networks^8^. Moreover, integrating fog computing has a great impact in a situation of foggy days. This benefit significantly influences scenarios involving foggy weather. Unpredictable weather can obstruct communication among vehicles. By combining fog computing with VANETs, data can be processed locally in these situations. This reduces reliance on remote servers and mitigates problems arising from unstable weather that lower network reliability^9^. Additionally, fog computing can effectively prevent traffic accidents on the road, improving driving conditions and potentially saving lives.

Vehicular fog computing (VFC) refers to the integration of fog computing within vehicular networks. In VFC, vehicles are utilized as part of the infrastructure to maximize the use of computing resources and facilitate vehicular communication. The primary objective of VFC is to leverage numerous nearby edge devices or end-user clients for processing tasks and communication activities.

VFC utilizes cloud characteristics, like providing applications, storage, and computing services to end-users. Furthermore, VFC distinguishes itself from existing models through its dense geographical spread and proximity to end customers.

**Fig. 2:**
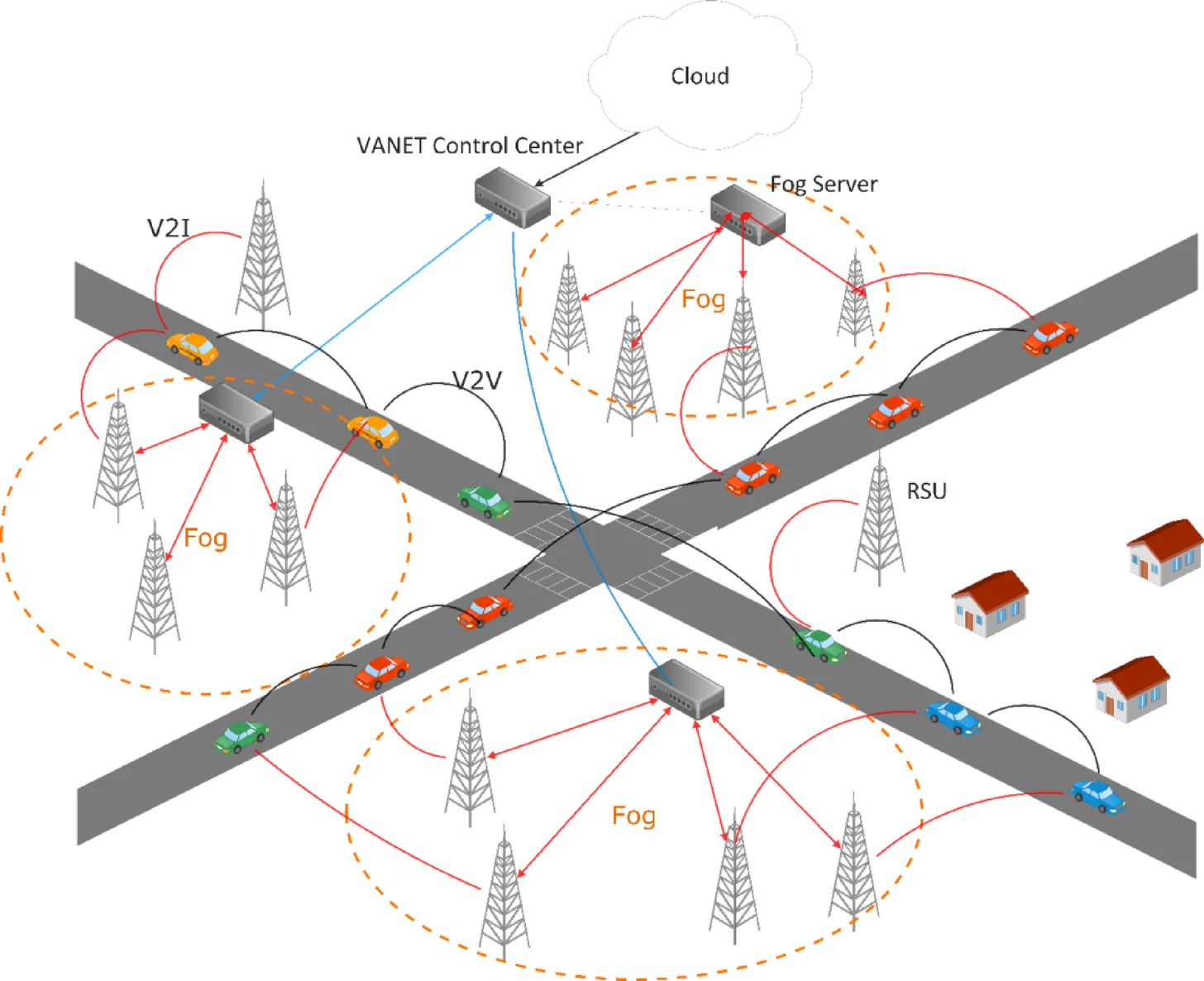
Layered fog computing architecture for VANETs.

Compared to other systems, VFC consistently delivers low latency in automotive communications. Figure 2 shows different communication types in fog-based VANETs. There are three types of connections made by fog servers in VANETs:

- RSU to Fog Server: The Fog Server receives information from RSUs in VANET, where V2V and V2R communication are utilized to spread safety and non-safety data. RSUs can also set up communication with one another, thereby serving as the backbone of fog. There is a wired/wireless connection between the RSU and the fog server.
- Fog Server to Fog Server: Fog servers in several locations manage a pool of resources for a specific area. Peer fog servers can communicate directly through wired or wireless connections, or they can be linked via a vehicular control center. It enables collaborative service provisioning and content distribution among peer fog servers, which improves overall system performance.
- Fog Server to Cloud: The cloud serves as the central controller for the fog servers that are distributed in multiple locations. Each fog server gives services to users in specified areas. Cloud aggregates the information received from fog servers and performs centralized computation, and fog servers convey the information received from the cloud to application users^10^.

Despite the numerous advantages of V2X communication, this communication presents completely new security challenges and difficulties. These challenges have never been addressed in a similar situation^11^. Besides, a vehicular transmission includes the location, velocity, and heading of the vehicle, making privacy a crucial concern in VANETs^12^. The wireless nature of communication between the onboard units (OBUs) of the vehicle and the roadside units (RSUs) makes it vulnerable to several known security threats. The quantity and variety of vulnerable wireless interfaces also rise as increasingly different sorts of entities. The new communication standards established for the vehicular network, DSRC and V2X, further raise the possibility of unidentified cyberattacks^13^.

 For the previous reasons, circumstances will be disastrous, especially if a safety message is issued. Consequently, several security solutions have emerged in recent years for OBU-RSU and OBU-OBU communication links in vehicular networks, utilizing advanced technologies like blockchain and artificial intelligence (AI) and cryptographic schemes^14^. It has been proven that these strategies can protect the network from a variety of cybersecurity attacks. Meanwhile, it is difficult to secure every possible link in an IoV network. And guarantee each entity’s privacy. For that, intrusion detection systems (IDSs) are needed to supplement these security plans.

Inter-vehicle network IDSs based on machine learning have also been proposed. However, there are two major challenges in the context of VANETs. Firstly, the incredible rate at which vehicle data is created. Secondly, the diversity of cyberattacks that are launched. These challenges make machine-learning approaches not feasible^15^.

Contrarily, deep learning does not require manual feature extraction, making it more appropriate for IDSs in the IoV networks. Vehicle OBUs have limited resources. Therefore, performing deep learning prediction tasks on them may be computationally inefficient. A deep learning architecture was proposed for detecting anomalies in 5 G-powered cellular networks, based on fog computing. According to this architecture, the RSUs gather the data transmitted by a vehicle. After that, RSUs send it to the fog servers that have deployed deep learning classifiers. The fog servers utilize one or more pre-trained deep learning models deployed as deep learning classifiers to identify several types of misbehavior in the incoming data. Hence, it decides the proper action. Various current papers classify vehicles by selecting certain features from their messages. This makes the classification schemes sometimes not perfect. Furthermore, a lot of other work doesn’t take all possible attacks into consideration. Thus, this paper aims to present a realistic VANET scenario. Vehicles are classified based on all features in the messages. Moreover, all misbehavior classes are classified using different classification models. We also differentiate between fault classes and attack classes. Finally, we compare the time and space complexity of all models used. Consequently, the major contributions of this paper can be summarized in the following points:

i. The development and comprehensive evaluation of two complementary classification frameworks. The first framework performs classification across all 20 classes available in the dataset (1 normal behavior class and 19 misbehavior classes) to detect intrusions in VANETs, taking into consideration all features of messages of a vehicle.
ii. The second framework provides classification into three broader categories:
  - Normal – the vehicle functions according to regular movement and communication behaviors.
  - Faulty – the vehicle displays irregular behavior resulting from internal sensor or system malfunctions (non-malicious).
  - Malicious – the vehicle purposefully tries to mislead or interfere with the network, signifying an attacker.
iii. A systematic comparison of multiple deep learning architectures under identical experimental conditions, enabling a fair assessment of their effectiveness for vehicular misbehavior classification.
iv. An analysis of computational efficiency, including execution time and resource requirements, to assess the suitability of the proposed models for fog-assisted VANET deployment.
v. The integration of the proposed classifiers within a conceptual fog-assisted intrusion detection framework, together with an analysis of their computational feasibility for real-time VANET applications.
vi. Improving recall, precision, and F1 scores compared to other papers.

The remainder of the paper is structured as follows: “Related work” section represents a brief about the sources of the papers used, “Proposed misbehavior classification scheme for intrusion detection” section lists the various models that were used to apply on the fog server in VANET, “Results” section lists the results, “Discussion” section represents a follow-up discussion on the performance and comparison with other work, “Conclusion” section presents the conclusion, and the last section lists the references.

## Related work

Numerous research studies and academic papers have addressed the subject of VANETs and the methods employed to tackle the challenges related to them. Researchers have dedicated significant effort to analyze, assess, and improve this framework as VANETs continue to be a prominent area of investigation. Two distinct categories of documents are examined:

1. The first category includes articles that discuss the integration of fog computing with VANETs,
2. while the second category concentrates on strategies for managing various types of attacks against it.

### Fog computing with VANETs

To maximize its potential, researchers focus on AI methods. Mchergui et al.^16^ provided a thorough analysis of the AI methods now being investigated by several research projects in the field of VANETs. They illustrated the advantages and disadvantages of these proposed AI-based methods for the VANET environment. They provided a table that lists the main advantages of the different machine learning algorithms. Moreover, they listed the different VANET applications where they could be most beneficial from these algorithms. The various applications, designs, technical problems, and important performance indicators using fog computing in-vehicle networks are the main topics of the review proposed by Behravanand et al.^17^. Researchers paid attention to reducing latency in VANETs. A classification of intelligence-based clustering algorithms, mobility-based algorithms, and multi-hop-based algorithms was presented by Mukhtaruzzaman et al.^18^. They examined the mobility metrics, assessment standards, challenges, and probable future paths of machine learning, fuzzy logic, mobility, NEMO, and multi-hop clustering algorithms. They used simulation models EdgeCloudSim and CloudSim. They proved the value of utilizing edge computing in conjunction with cloud computing. Combining edge computing with 5G for transportation networks improved the system. They proved that this architecture minimized delay and processing time. While edge computing is not a substitute for cloud networks, it enables processing and communication with minimal latency. Mscs et al.^19^ developed a fuzzy logic-based task scheduling system in VANET aimed at reducing latency and enhancing response time for task offloading in the IoV. This method efficiently allocated workloads to the fog computing layer, considering the limited resources of vehicle nodes. Through simulation tests, they demonstrated the effectiveness of the proposed task scheduling framework, surpassing existing algorithms and successfully minimizing average latency time within the network parameters.

A lot of research was introduced to show the importance of using edge computing in VANETs. Patil et al.^20^ suggested a way to assess the effectiveness of edge and cloud technologies based on several indicators. They tried to identify the approaches most suited for deployment in automotive networks. The outcome proved that edge computing results in a 62% reduction in latency and a reduction in server usage time. Furthermore, network performance is crucial. So, Hamayoun Shahwani et al.^21^ thoroughly assessed the literature on current data distribution approaches in VANET. They began by outlining the categories of significant concerns that may be addressed for data gathering in VANET and then explained the current techniques for disseminating data.

Furthermore, researchers tried to enhance fog-based VANETs. Shahin et al.^22^ devised a cost-effective plan to address the localization and configuration issues with fog-based RSU deployment in VANETs. According to the amount of computational demand inside each fog node’s coverage zone, the suggested technique could allocate each fog node’s computational capability. They planned to distribute and build up a group of fog-based RSUs to meet demands and application-specific requirements.

Additionally, researchers studying VANETs pay close attention to safety applications. Some of them dealt with the difficulties of spreading safety messages to cars. In addition, Yu et al.^23^ proposed a federated learning-driven edge caching approach to meet the requirements of future automotive networks. These networks demand low latency and high computation capabilities for various applications. A method for addressing the needs of high mobility, user privacy, and scalability in vehicle networks was proactive content caching at the network edge.

These studies all focused on network performance and lowering latency. Other efforts have focused on how to spread messages solely from reliable sources. Chen et al.^24^ presented a framework for vehicular edge computing to incorporate deep reinforcement techniques into an intelligent path-planning strategy for platoons of autonomous vehicles. In several additional research studies, deep Q-learning and edge computing were coupled to produce effective vehicle caching. Meanwhile, they expected the network to be secure. They also supposed all vehicles acted well without any possible faults. Therefore, a lot of research has paid attention to possible attacks on VANETs.

### Machine and deep learning approaches for attack and fault detection in VANETs

There are several studies to address the problem of several faults and attacks that could exist in VANETs. Some of them used machine learning techniques, and the others used deep learning. The summary of the related studies is presented in Table 1.

#### Machine learning-based intrusion detection techniques

Setia et al.^26^ proposed a novel approach to detecting DDoS attacks in the context of vehicular ad hoc networks (VANET Cloud). Notably, this study was the first to investigate machine learning-based approaches for detecting DDoS attacks in VANET cloud systems. This investigation made a significant contribution by conducting extensive statistical analyses of network traffic characteristics, which included both normative network conditions and adversarial attack scenarios. This careful analysis has helped to develop critical criteria and thresholds for accurately identifying potential DDoS attack risks. However, the proposed work scored low in accuracy and recall. It also introduced high latency. Bangui et al.^31^ presented a novel machine learning approach that enhances IDS performance. They achieved this by integrating Random Forest with posterior detection based on coresets, resulting in improved detection accuracy and efficiency. The experimental results indicated that the suggested machine learning model significantly improved detection accuracy compared to traditional methods.

There was research that addressed specific types of attacks. Kakulla et al.^32^ put forward the SDTC (Sybil node detecting the use of Classification) strategy, which depended entirely on machine learning methods to thwart Sybil attacks on VANETs. To minimize identification time, raise detection accuracy, and improve scalability, the SDTC mechanism employed a few vehicle-specific Extreme Learning Machine (ELM) characteristics. The findings implied that SDTC is a proper method for reducing Sybil attacks and maintaining provider service in VANETs. Chakraborty et al.^27^ used machine learning to suggest a unique security technique for smart transportation that increases communication and intruder detection in VANET. Ciphertext-policy game theory encryption analysis for smart transportation was applied in this case to improve VANET security. Fuzzy rule-based encoder perceptron neural networks were used to detect VANET intruders. An experimental study was conducted on a variety of network datasets in terms of throughput, QoS, latency, computational cost, and data transmission rate. The use of small datasets is a common problem in these publications. To deal with large datasets, researchers turn to deep learning systems.

#### Deep learning approaches for misbehavior classification in VANETs

Recent studies have investigated the deployment of lightweight deep-learning-based intrusion detection systems in fog environments. For example, Mohapatra et al.^35^ proposed a CNN-LSTM-based IDS optimized for fog computing, demonstrating that combining convolutional and recurrent layers can achieve high detection accuracy while maintaining reasonable computational requirements for edge deployments.

M, M.V.B. et al.^28^ described an effective invasive weed optimization algorithm based on energy-efficient clustering and deep wavelet neural network-based intrusion detection (IWOEEC-DWNN) technique for VANET. The IWOEEC-DWNN technique targeted the clustered VANET and aims to detect any potential intrusions. Tejasvi Alladi et al.^34^ proposed a deep neural network-based vehicle anomaly detection scheme. They used a sequence reconstruction approach to differentiate normal vehicle data from anomalous data. Numerical results showed that they could correctly detect data corresponding to several anomaly types. However, they dealt with all misbehavior as attacks.

However, Chen et al.^36^ introduced VAN-FED-IDS, a federated learning-based intrusion detection framework that integrates Bi-LSTM and LightGBM through Dempster–Shafer fusion to improve privacy preservation and accelerate distributed model updates in VANETs. The framework utilizes RSUs for local training and cloud-based aggregation, demonstrating high detection accuracy for DoS and Sybil attacks while reducing the need to share raw data. Nevertheless, the study evaluates single hybrid architecture and focuses on specific attack scenarios, without investigating the impact of different deep learning architectures on multi-class misbehavior classification under identical experimental conditions. Gurjot Kaur and Deepti Kakkar^33^ created a hybrid optimization-based Deep Maxout Network (DMN) for attack categorization in VANET. The Cluster Head (CH) selection and routing operation was carried out utilizing a hybrid optimization technique. Performing an accurate classification procedure requires careful consideration of the feature selection process. Furthermore, the attack classification is conducted using DMN, which was taught by a proposed optimization technique. The created optimization-based DMN model improved classification performance with a precision and recall of 0.9395 and 0.9462, respectively. Conversely, they failed to consider a data-driven intrusion detection approach to provide automatic security services.

Deploying deep learning networks within localized intelligent transportation systems introduces distinct trade-offs between architectural accuracy and edge network resources. As thoroughly surveyed by Mohapatra^37^, edge and fog-computing environments are strictly bound by tripartite operational constraints involving energy capacity, processing latency, and security exposure during network failover windows. Consequently, implementing heavy frameworks at the edge mandates a clear partitioning of tasks, ensuring that core computation is optimized to avoid breaching the strict real-time throughput budgets inherent to decentralized infrastructure.

Federated learning plays a vital role in intrusion detection systems. Huang et al.^38^proposed FED-IoV, a federated learning-based intrusion detection framework for the Internet of Vehicles (IoV) that addresses both computational efficiency and data privacy. The framework transforms vehicular communication traffic into image representations and employs a lightweight MobileNet-Tiny model for intrusion detection, while federated learning enables collaborative model training without sharing raw data. The proposed method was validated using the CAN-Intrusion and CICIDS2017 datasets and demonstrated high detection accuracy with low inference latency on resource-constrained devices such as Raspberry Pi, highlighting its suitability for edge-based IoV environments. However, the framework focuses primarily on lightweight image-based intrusion detection within a federated learning setting and evaluates a single deep learning architecture. It does not investigate the comparative performance of multiple deep learning architectures for multi-class vehicular misbehavior classification or distinguish between malicious attacks and non-malicious vehicle faults. Moreover, Taheri et al^39^. proposed an Explainable Federated Learning-based Intrusion Detection System (XAI-FL-IDS) for connected vehicles that integrate a Deep Neural Network (DNN) with Federated Learning (FL) and SHAP-based Explainable Artificial Intelligence (XAI). The proposed framework enables collaborative model training without sharing raw vehicular data, thereby preserving privacy while improving the transparency of intrusion detection decisions. Experimental evaluation on the CICEVSE2024 and CICIoV2024 datasets demonstrated high detection accuracy together with enhanced interpretability and resilience compared with conventional federated IDS approaches. However, the framework focuses on explainability and privacy-preserving distributed learning using a single DNN architecture, without investigating the comparative effectiveness of different deep learning architectures for multi-class vehicular misbehavior classification or analyzing their computational complexity for fog-assisted deployment.

Tripathi et al.^29^ suggested a modified deep metric learning-based chaotic Henry gas solubility optimization (MDML-CHGSO) method for developing trust-based security designs. The proposed trust model was based on plausibility and previous experience protecting vehicle networks. The suggested model used multiple methods to verify the legitimacy of the received data from the approved vehicle, including authentication, lifespan verification, experience appraisal, plausibility determination, and accurate measurement. The updated CHGSO technique, based on DML, accurately recovered the position of the event message when it is near the fog nodes. In addition, the measurements used to rate performance include average latency, throughput, and packet delivery ratio. Despite all these achievements, the additional processing required for deep metric learning might still introduce delays in real-time applications. Datasets are a key factor in the context of using different classification models. One of the most used datasets is the VeReMi extension dataset. It has 20 different types of vehicles. Saudagar et al.^30^ proposed a novel deep learning technique for detecting location falsification attacks in VANETs and uncovering hidden patterns in data to combat known and emerging threats. It has a high detection accuracy of 99.55% for diverse attacks. On the other hand, they didn’t consider the fault types of vehicles. Setitra et al. in^25^ provided a deep learning technique for recognizing DDoS attacks on SDN-based VANET, also known as TabNet. To improve its performance, the model was tuned using hyperparameters and Adam optimization. It reached an overall accuracy of 99.42%. However, the approach relied heavily on SDN, which introduces a single point of failure.

Despite recent advances in deep learning-based Intrusion Detection Systems (IDS) for VANETs - particularly hybrid architectures like CNN-LSTM- three critical limitations remain in existing literature:

1. Most studies evaluate only a single network architecture. Consequently, it is difficult to determine which architecture provides the best trade-off between detection performance and computational efficiency under identical experimental conditions.
2. Many studies focus on detecting a single attack type (e.g., DDoS or Sybil), limiting their applicability to comprehensive misbehavior detection.
3. The vast majority of recent deep learning methods exclusively target malicious cyber-attacks, failing to differentiate between intentional attacks and non-malicious operational vehicle faults.
4. Existing approaches frequently train on reduced feature subsets and omit formal algorithmic complexity analyses (time/space complexity), offering no guarantee of operational feasibility in edge-fog environments.

To address these limitations, this work proposes two complementary classification frameworks. The first performs fine-grained classification across all 20 behavior classes in the VeReMi Extension dataset, whereas the second classifies vehicle behavior into three practical categories (Normal, Faulty, and Malicious). Furthermore, four deep learning architectures are systematically evaluated under identical preprocessing, training, and testing conditions to ensure a fair comparison. In addition to classification performance, this work analyzes execution time and computational complexity to identify the most suitable model for real-time fog-assisted VANET deployment.

Table 2 presents a comparison of performance metrics from the studies examined in this literature review alongside the two classifiers.

**Table 1:** Summarization of related work studies.

Reference	Year	Category	Approach	Pros	Cons
Setitra et al.^25^	2024	Deep learning	Provided a deep learning technique for recognizing DDoS attacks on SDN-based VANET, also known as TabNet.	High accuracy and reduced false positives. Suitable for large-scale VANET deployments	Single point of failure Limited to DDoS attacks
Setia et al.^26^	2024	Machine learning	Detecting DDoS attacks in the context of VANET Cloud.	The creation of crucial standards and parameters for the precise detection of any DDoS attack threats	Kernel SVM scores extremely low accuracy and recall Limited to DDoS attacks
Chakraborty et al.^27^	2023	Machine learning	Vehicles are divided into clusters, each managed by a roadside unit. Before accessing vehicle ad hoc networks, the RSU validates node credibility.	More than a network scenario Successfully reducing the number of attack nodes in VANET, demonstrating its ability to detect and remove them over time	High latency; evaluation focuses mainly on detection performance without a detailed comparison of alternative deep learning architectures
M, M.V.B. et al.^28^	2023	Deep learning	Describe an effective invasive weed optimization algorithm based on energy-efficient clustering and a deep wavelet neural network-based intrusion detection technique to detect any potential intrusions.	Higher efficiency with the increase in vehicle	Applicability to heterogeneous attack types and computational overhead in fog environments is not comprehensively discussed
Tripathi et al.^29^	2023	Deep learning	modified deep metric learning-based chaotic Henry gas solubility optimization approach for creating a trust-based security design	High performance in terms of average latency, throughput, and packet delivery ratio	The additional processing required for deep metric learning might still introduce delays in real-time applications. Does not compare multiple deep learning architectures under identical settings
Saudagar et al.^30^	2023	Deep learning	proposed a novel deep learning technique for detecting location falsification attacks in VANETs	High accuracy	Evaluates a single IDS framework. Comparative architectural analysis and computational complexity evaluation are limited
Bangui et al.^31^	2022	Machine learning	A new machine learning model to improve IDS performance by combining Random Forest with posterior detection based on coresets to improve detection accuracy and efficiency.	increasing the detection efficiency and decreasing computational time	Old dataset, Limited to DDoS attacks, Evaluates a single hybrid CNN–LSTM architecture, with limited architectural comparison.
Kakulla et al.^32^	2022	Machine learning	Reducing Sybil attacks and maintaining provider service in VANETs.	High detection rate with a significantly reduced number of errors.	Limited to Sybil attacks
Gurjot Kaur and Deepti Kakkar^33^	2022	Deep learning	A hybrid optimization-based Deep Maxout Network for attack categorization in VANET	Improved precision and recall in different models. Work on 2 different datasets	Failed to consider a data-driven intrusion detection approach to afford automatic security services
Tejasvi Alladi1 et al.^34^	2021	Deep learning	A deep neural network-based vehicle anomaly detection scheme	Correct detection of data corresponding to several anomaly types	They dealt with all misbehavior as attacks

**Table 2:** Summary table for comparing performance metrics.

Study	Method	Dataset	Precision %	Recall%	F1-score%	Limitations
Setitra et al.^25^	Custom SDN-based VANET dataset	SDN-based VANET	98.5	98	97.6	Focuses on SDN-based VANETs
Setia et al.^26^	ML-based DDoS Detection	VANET Cloud	96.8	97.5	97.1	high computational cost
Bangui et al.^31^	Hybrid ML Model	NSL-KDD, UNSW-NB15	93.2	94.1	93.6	Requires large training data
Kakulla et al.^32^	ML for Sybil attack detection	Custom VANET dataset	93.5%	94.7%	94.1%	A small dataset size may lack diversity in attack scenarios.
Chakraborty et al.^27^	ML-based Secure Framework	Custom VANET dataset	96.5	97.3	96.9	Focuses on specific attack types
M, M.V.B. et al.^28^	Deep Wavelet Neural Network	Clustered VANET dataset	92.7	93.4	93	may not scale well for large networks.
Tejasvi Alladi1 et al.^34^	Deep Neural Networks	IoT-VANET Dataset	97.2	97.8	97.5	Requires large training data
Gurjot Kaur and Deepti Kakkar^33^	Deep Learning for Attack Detection	Custom VANET dataset	94.8	95.6	95.2	Complex model
Tripathi et al.^29^	Deep Metric Learning	Custom fog-based VANET dataset	96.1	96.8	96.4	Limited generalizability
Saudagar et al.^30^	VeReMiNet IDS	VeReMi Dataset	92.9	93.6	93.3	Low results compared to other studies
Proposed work _First classifier	Deep Learning	VeReMi extension dataset	97.2	95.8	95.9	
Proposed work_ Second classifier	Deep Learning	VeReMi extension dataset	99.7	99.6	99.8	

## Proposed misbehavior classification scheme for intrusion detection

### Network model

**Fig. 3:**
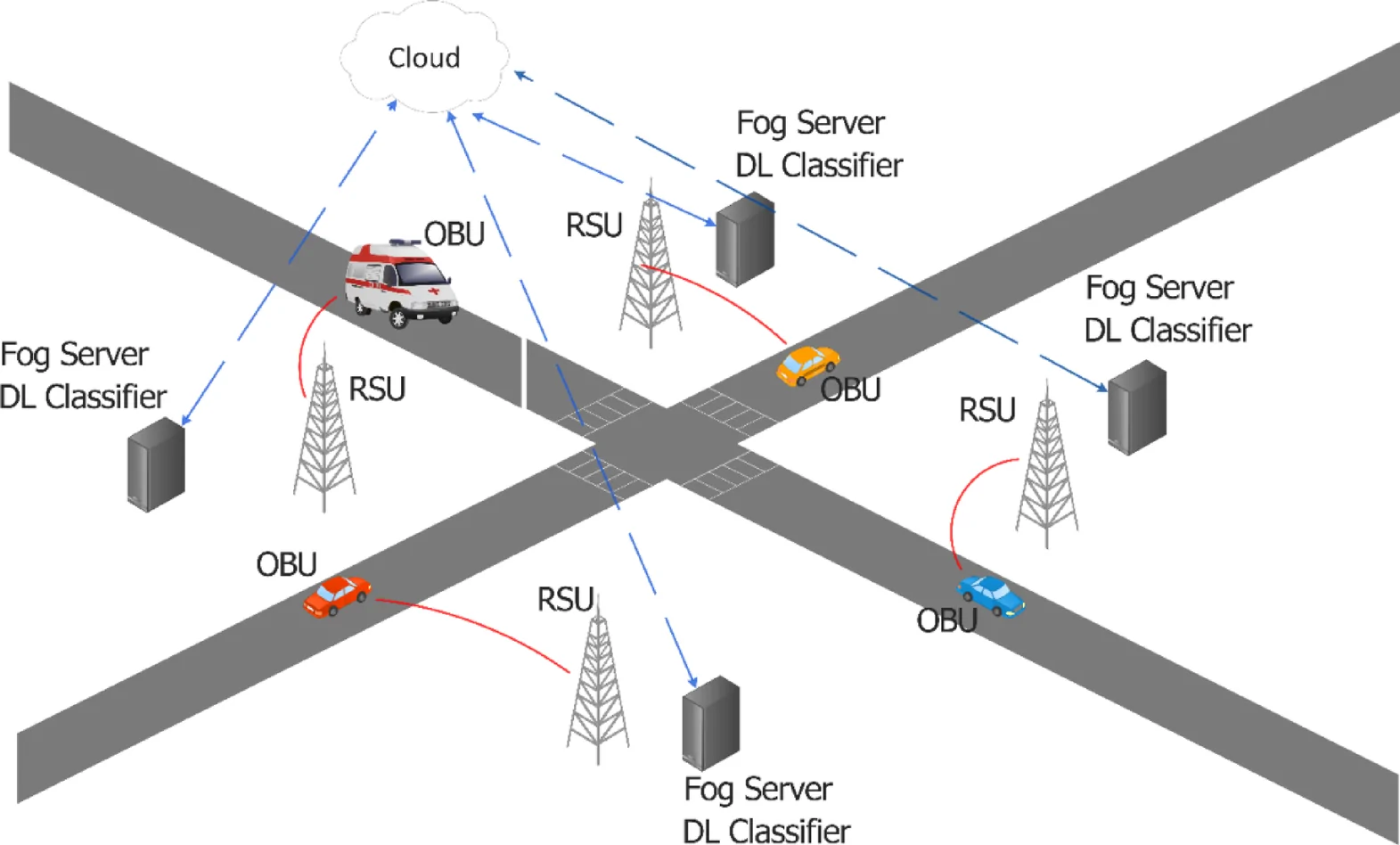
Proposed system network model.

Consider a group of vehicles sharing information with RSUs stationed at the intersections of roads. The car devices can communicate with the roadside units using special communication technology. Every roadside unit in the network is located together with a server at the edge, where the deep learning models are installed. The fog servers are linked to the cloud servers using fast-wired connections. While the actual vehicle misbehavior prediction tasks are conducted on the fog servers, the deep learning models are developed on cloud servers. When data is transmitted by a vehicle, it is received by the nearest RSU. The data is then sent by the RSU to the edge server to check for any improper behavior. The network model depicted in Fig. 3 illustrates this process.

By integrating fog computing into VANETs, the proposed framework can adapt to different environmental situations, including challenging weather conditions like rain, fog, or snow. In these scenarios, sensor data may become unreliable, delayed, or even partially lost, which can impact the quality of information gathered from vehicles.

Fog nodes, located nearer to the network’s edge, facilitate real-time data filtering, aggregation, and preprocessing, which improves the dependability of the input data before it is sent to the classification model. This local processing reduces the adverse effects of environmental disturbances.

Additionally, the deep learning model has been trained on a wide range of behavioral patterns that can represent both sensor malfunctions and unpredictable driving behaviors, some of which can be indirectly affected by weather. This setup enables the model to distinguish between erroneous sensor readings caused by environmental factors and deliberate malicious actions, thus ensuring strong detection performance in difficult operating conditions.

The cars could behave in a normal manner or exhibit several types of misbehavior. Misbehavior can be categorized into different faults or attacks. This study utilized data from the VeReMi Extension, which is a popular dataset for detecting misbehavior in vehicular networks^40^. This dataset includes a variety of kinds of misbehavior, including cyberattacks like Denial of Service (DoS) attacks, replay attacks, and their variations^41^, as well as errors relating to wrong location and velocity measurements. According to the behavior of the vehicle, action is taken.

### Data preprocessing

#### Structure of the dataset

There are 24-hour simulations in the VeReMi Extension dataset, one for each hour of the day from hour 0 to hour 23. Each simulation consists of one Ground Truth (GT) file comprising all the data that was received by the fog servers during that hour, combined with the original trace logs of each vehicle that traveled through the network during that hour. Send time, sender ID, sender pseudoID, message ID, and position-based information, such as location, velocity, acceleration, and direction, as well as the noise in each of these variables, are just a few of the fields that make up the data in these log files and the GT file of each simulation. The 24 GT files were utilized since the study focused on misbehavior detection at the level of fog servers.

**Fig. 4:**
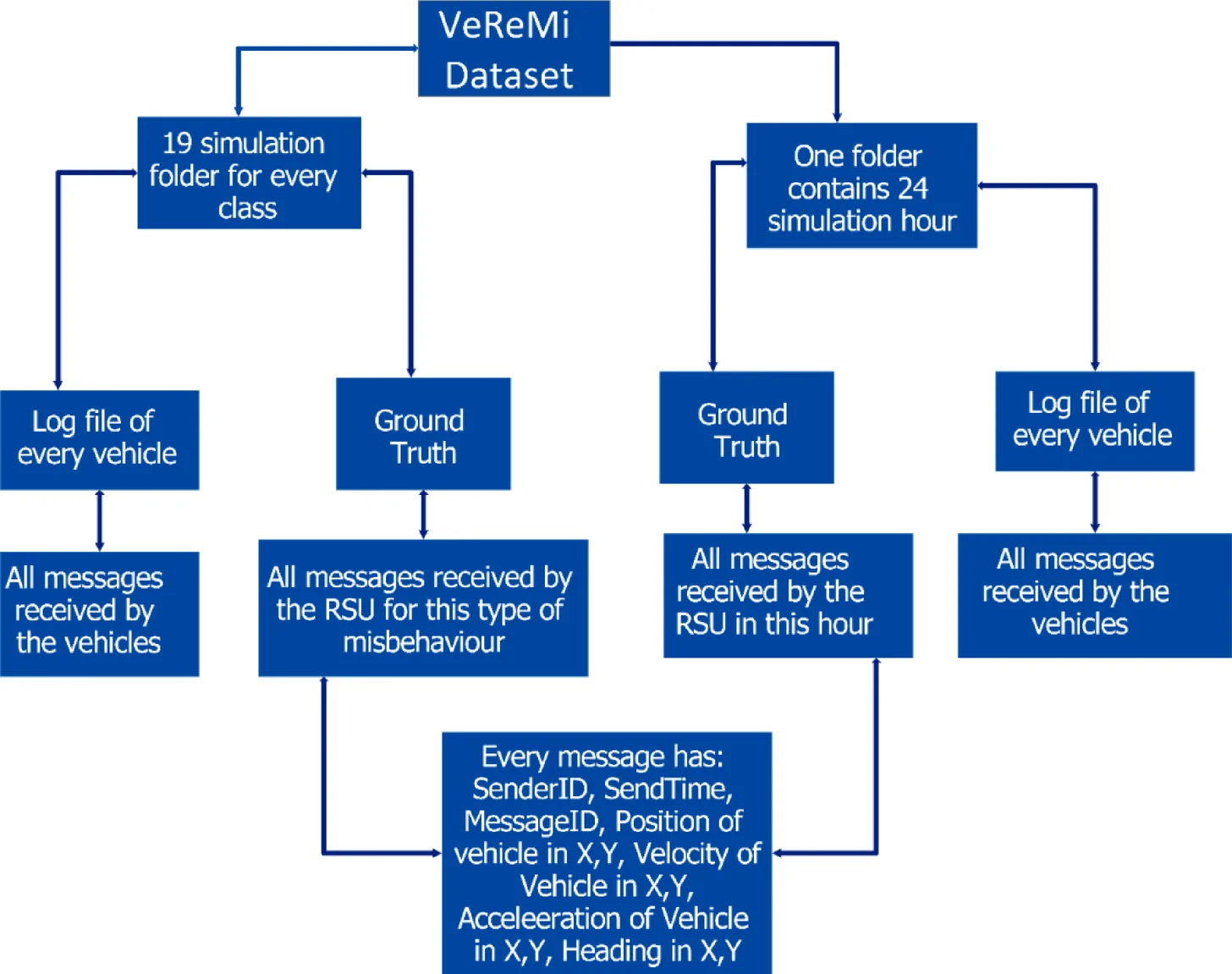
illustrates the full structure of the dataset.

Figure 4 Hierarchical structure and feature details of the VeReMi dataset.

#### Field selection and labeling

Sender ID, sender pseudoID, send time, two X and Y coordinate values of each position, velocity, acceleration, and direction, among other data fields provided in the GT file, are necessary for our investigation. The vehicle entries provided in the GT files do not have any misbehavior labels attached to them. The vehicle-wise log files for the relevant simulation hour provide this information. All the simulated hours’ GT files were studied. Then, it was chosen to select all the features and provide misbehavior labels from the log file names for each given vehicle to generate a simplified vehicle-wise dictionary that contained the labels and data required for each.

#### Sequence creation

Several experiments were conducted to choose the optimal sequence length. When larger sequence lengths (greater than 20) were tested, the number of generated sequences decreased substantially for several minority classes. As a result, some classes contained insufficient samples for effective training and evaluation, while others became severely underrepresented. This negatively affected class coverage and increased class imbalance within the generated sequence dataset.

Conversely, smaller sequence lengths were also examined. Although they increased the number of generated sequences, they significantly increased the computational cost and training time without providing a noticeable improvement in classification performance.

Based on these observations, a sequence length of 10 was selected as a practical compromise between preserving class representation, maintaining computational efficiency, and achieving strong classification performance.

**Fig. 5:**
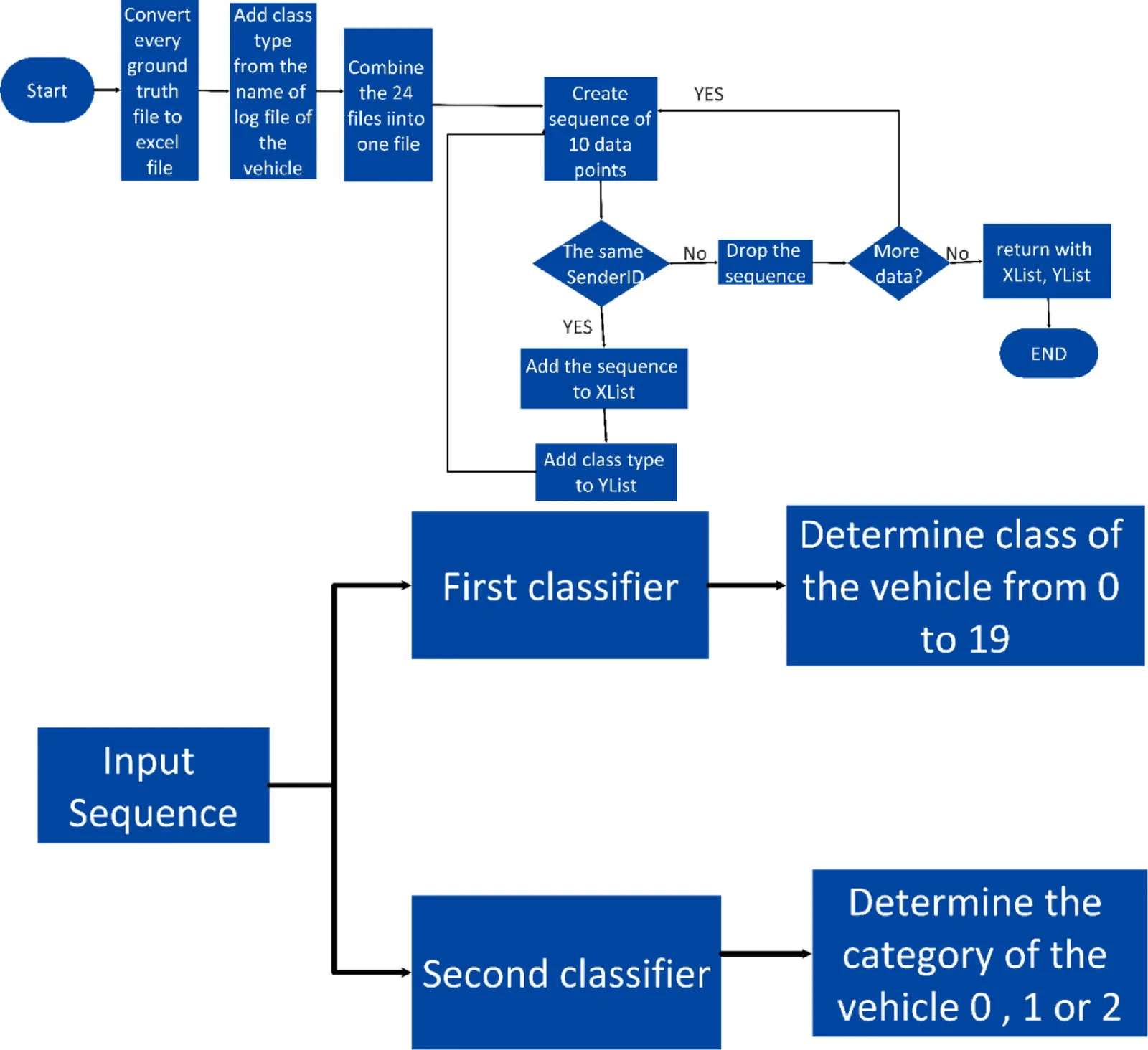
Flow chart of preprocessing.

The reduced vehicle data dictionary was used to produce several time sequences, each with ten data points. Label, send time, pseudoID, X and Y location coordinates, X and Y position noise coordinates, X and Y velocity coordinates, X and Y velocity noise coordinates, acceleration and heading coordinates, and their noise in the coordinates of X and Y. All are the variables that make up each data point. Sequences shorter than ten were not taken into consideration. Once all a vehicle’s time sequences have been created, they are added to the dictionary of misbehavior class as shown in Algorithm 1. Figure 5 shows the flow chart of the preprocessing of data.

**Algorithm 1:**
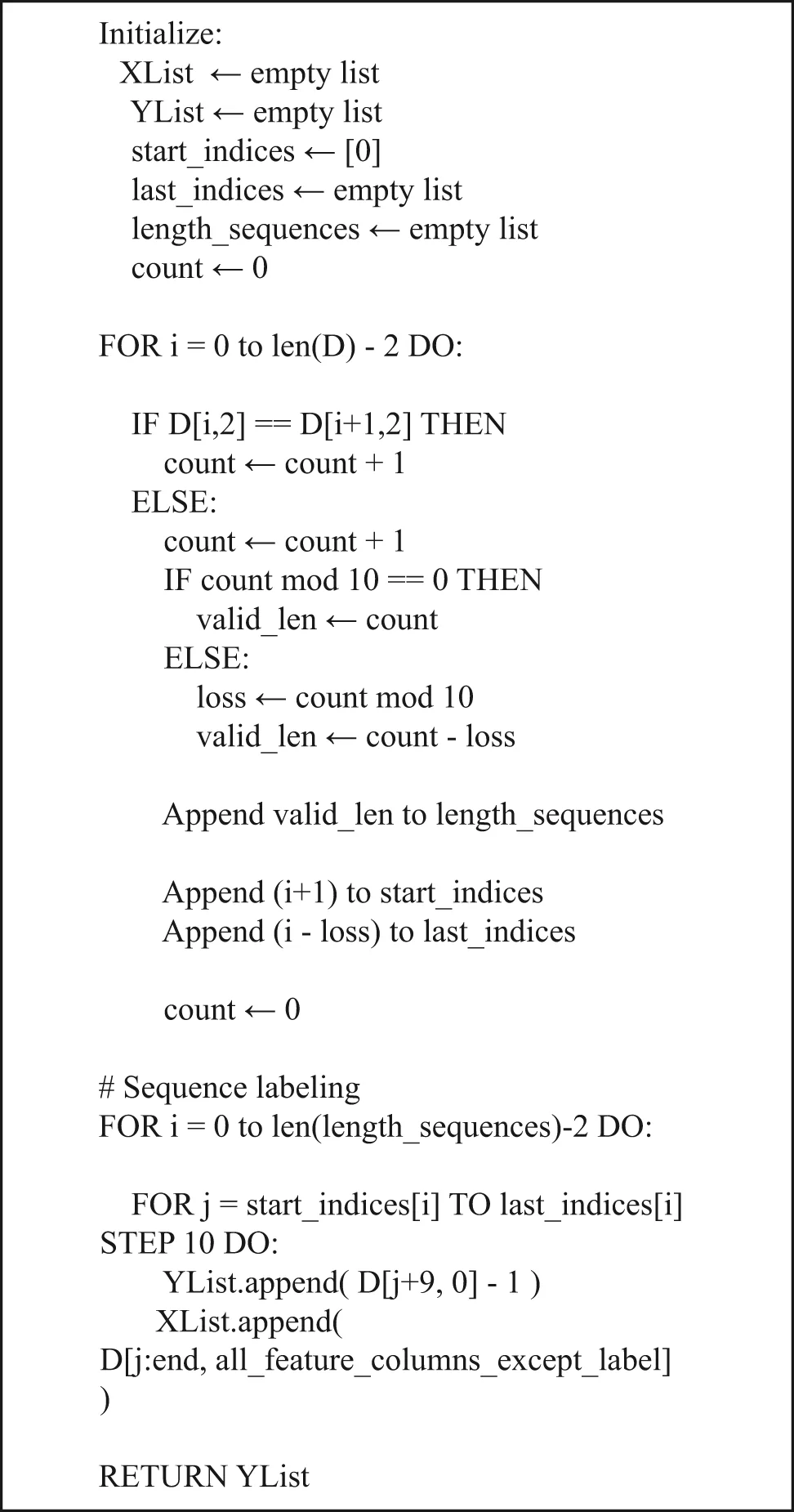
Preprocessing of data.

#### Misbehavior class selection

In this study on classification, the goal is to classify twenty different class types from the original VeReMi Extension dataset. As seen in Table 3, this contains one normal vehicle behavior type, nine fault kinds, and ten attack types. The number of generated sequences for each behavior class after sequence construction, illustrating the class distribution used throughout the experiments.

**Table 3:** Classes and their sequences.

Type	Class	Description	Total seq.	Percentage (%)
0	Normal	Normal behavior	43,433	59%
1	Fault	Constant position	903	1%
2	Fault	Constant position offset	989	1%
3	Fault	Random position	991	1%
4	Fault	Random position offset	959	1%
5	Fault	Constant speed	950	1%
6	Fault	Constant speed offset	937	1%
7	Fault	Random speed	916	1%
8	Fault	Random speed offset	1004	1%
9	Fault	Delayed Messages	957	1%
10	Attack	Disruptive	1031	1%
11	Attack	Data replay	990	1%
12	Attack	DoS	881	1%
13	Attack	DoS random	3348	5%
14	Attack	DoS disruptive	3067	4%
15	Attack	Data replay sybil	3091	4%
16	Attack	Traffic congestion sybil	4122	6%
17	Attack	DoS random sybil	1027	1%
18	Attack	DoS disruptive sybil	1966	3%
19	Attack	Eventual stop	1913	3%

The abnormal behaviors observed in the dataset are categorized into two separate groups: fault classes and attack classes, depending on the nature and purpose of the deviation.

Fault Classes indicate unintended, non-malicious irregularities that often arise from sensor failures or calibration mistakes. These faults impact both positional and velocity data:

- Positional faults: where a vehicle inaccurately reports its location with either constant or random discrepancies, either holding a steady position or changing it erratically.
- Velocity faults: where a vehicle alters its speed by introducing constant or random changes to its actual velocity, replicating unusual movement patterns.

Attack Classes consist of deliberate, malicious actions intended to disturb the network or deceive other nodes. These encompass:

- Denial of Service (DoS): overwhelming the network with an excessive number of messages, denying vehicle nodes the ability to access the infrastructure’s operational capabilities.
- Sybil Attacks: employing numerous fake identities (pseudoIDs) to exert inappropriate influence in the network. Due to the authenticity of the IDs, identifying such misconduct is challenging.
- Data Replay: retransmitting previously recorded legitimate data from another vehicle, complicating detection due to the utilization of authentic patterns.
- Disruptive Attacks: A variant of data replay where messages from several vehicles are randomly broadcast, leading to network congestion.
- Eventual Stop Attack: The vehicle halts its position and adjusts its velocity to zero, creating the illusion of an abrupt and unrealistic stop.

Table 4 presents the key differences between fault and attack classes.

**Table 4:** Comparison between fault and attack classes.

Aspect	Fault classes	Attack classes
Definition	Unintentional errors (due to internal system or sensor faults)	Malicious actions aimed at disrupting network operations.
Cause	Sensor failure, noisy data, or GPS deviation	Malicious individuals are taking advantage of weaknesses (e.g., impersonation, overwhelming traffic).
Examples	Constant position Random position Constant velocity Random velocity	Data replay attack Sybil attack Disruptive replay DOS attack
Detection difficulty	Moderate (patterns may show inconsistencies, but can be identified through statistical techniques)	High (certain attacks replicate normal activity or utilize valid IDs, complicating detection).

### Deep learning classifier

Deep learning imitates the human brain’s ability to assimilate data and develop patterns from it to make decisions, and it can learn from unstructured and unlabeled data. Deep learning models include Multi-Layer Perceptron (MLP), Convolutional Neural Network (CNN), Recurrent Neural Network (RNN), and Long Short-Term Memory (LSTM).

A CNN is a hierarchical system that applies a sliding filter with a defined width to the input of each convolutional layer. After each epoch, the sliding filter adjusts to capture new observations. The input may be one dimension (1D) or more. In the present investigation, we use 1D convolutional layers. A CNN consists of an input layer, many hidden layers, and an output layer. The hidden layers consist of convolutional layers followed by normalization, pooling, or fully connected layers.

LSTMs are a sort of RNN that retains sequential information. RNNs outperform ANNs on sequential data, but they also suffer from vanishing gradient issues. This impairs the network’s ability to remember information over long periods of time since any two critical events in the time series may occur with large gaps. LSTMs are designed to tackle the problem of disappearing gradients by being somewhat insensitive to the gap length. Because of their ability to recall values across arbitrary time intervals, LSTM networks are ideal for classification issues involving time series data.

Because of the time-series structure of vehicle data, CNN and LSTM are the ideal deep learning models. Furthermore, combining both techniques will be quite beneficial in maximizing their respective benefits. Thus, the CNN and LSTM models are used in this study to classify misbehavior in sequential vehicular data.

We describe here two alternative types of classifiers for classifying and detecting potential intrusions in the IoV network.

#### First classifier

It is a classifier in which the deep learning model predicts and classifies the input sequence as normal, one of the eight fault categories, or one of the eleven assault types determined.

#### Second classifier

It is a classifier in which the deep learning model predicts and classifies the input sequence as normal, fault, or attack type.

### Training and testing

For all training of the deep learning models, “categorical_crossentropy” was chosen as the loss function as it is a multiclass classifier. The Adam optimizer is used with a learning rate of 0.0003. The dense layers are activated with the “softmax” activation function. Accuracy, precision, recall, and F1-score are defined in Eq. (1), Eq. (2), Eq. (3), and Eq. (4), respectively.

1 $$\mathrm{Accuracy} = \frac{TN+TP}{TN+FP+FN+TP}$$

2 $$\mathrm{Precision}= \frac{TP}{TP+FP}$$

3 $$\mathrm{Recall}= \frac{TP}{TP+FN}$$

4 $${\mathrm{F}}1_{\text{Score }}= 2* \frac{Precision*Recall}{Precision+Recall}$$

## Results

A series of experiments were carried out to arrive at the current configuration. To guide these experiments, we adhered to certain principles. First, due to the complexity of the dataset and the variety of features, a stacked model with varying layer counts, ranging from 3 to 4 layers for each model, was chosen. Second, recognizing that the dataset involves time series classification, a larger number of units of LSTM layers is opted for. Furthermore, standard starting points applicable to complex and extensive datasets are followed. Powers of 2 for the number of filters are selected for improved memory and computational efficiency.

All deep learning models were created using Keras with a TensorFlow backend, operating on a system that has an Intel Core i7 processor, 8 GB of RAM. The training of the models utilized the “categorical_crossentropy “loss function, which is appropriate for tasks involving multi-class classification. In each model’s output layer, the “softmax” activation function was employed. Dropout regularization of rate.3 was applied in the proposed architectures to reduce overfitting by randomly deactivating a fraction of neurons during training, which improves generalization ability.

Unless mentioned otherwise, the Adam optimizer was used with a learning rate set at 0.0003, and the training process spanned 50 epochs with a batch size of 100. The data was organized into sequences consisting of 10-time steps for each vehicle, and all features underwent normalization through MinMax scaling. The dataset was split into 75% for training and 25% for testing. A vehicle-level splitting strategy was adopted, where all sequences belonging to a given vehicle are assigned to either the training or testing set exclusively to avoid data leakage. The selection of this specific split, along with the network’s design settings and training time, was based on a careful set of early experiments. These initial tests tracked loss stabilization and convergence behavior. This ensured that the models could generalize well without overfitting. As a result, the final deep learning classifiers were tested directly on the independent 25% testing set. This verified their strength on new sequential data. Table 5 represents a summary of experimental settings.

**Table 5:** Summary of experimental settings.

Parameter	Value
Framework	Python 3.11 with Keras with TensorFlow backend
Hardware	Intel Core i7 CPU, 8 GB RAM
Loss function	Categorical crossentropy
Input dimensions	(10,21) (21 feature × 10 points)
Optimizer	Adam (in 4 LSTM, 2 CNN- 1 LSTM) and Adamx in (2 CNN- 2 LSTM, 3 CNN- 1LSTM)
Learning rate	0.0003
Batch size	100
Epochs	50
Kernel size	1
padding	valid
Output activation	Softmax
Normalization	MinMax Scaling
Data Split	75% − 25%

### First classifier

#### 4 LSTM

This model is designed to capture deep temporal dependencies across the input sequences. It consists of four LSTM layers with 256, 128, 64, and 32 hidden units, respectively. All LSTM layers utilize the tanh activation function. To ensure proper information flow through the recurrent stack, the first three LSTM layers have their return_sequences parameter set to *True*, whereas the final LSTM layer sets it to *False* to flatten the temporal output. Finally, a fully connected (Dense) output layer with a Softmax activation function is mapped to the target classes.

#### 2 CNN- 2 LSTM

This hybrid architecture integrates spatial feature extraction with temporal sequence modeling. The network begins with two 1D Convolutional (Conv1D) layers with 512 and 256 filters, respectively, utilizing the *tanh* activation function to extract localized spatial patterns. The output is then passed to a recurrent network consisting of two LSTM layers, each with 128 hidden units and a *tanh* activation function. The first LSTM layer has its return_sequences parameter set to *True*, while the second (final) LSTM layer sets it to *False*. This model is optimized using the Adamax optimizer instead of the standard Adam. The network concludes with a Softmax Dense layer for classification.

#### 3 CNN-1LSTM

This network employs a deeper convolutional hierarchy followed by a shallow recurrent structure. It comprises three sequential Conv1D layers with 512, 256, and 128 filters, respectively, to capture hierarchical spatial representations, all configured with a *tanh* activation function. The extracted spatial features are subsequently fed into a single LSTM layer with 64 hidden units and a *tanh* activation function, with its return_sequences parameter set to *False*. Similar to the previous configuration, the Adamax optimizer is utilized for network training, and a standard Softmax Dense layer is applied at the output.

#### 2 CNN-1 LSTM

This architecture provides a streamlined hybrid configuration. It features two sequential Conv1D layers, each containing 256 filters with a *tanh* activation function to process the input feature vectors. The output of the convolutional block is forwarded to a single LSTM layer with 128 hidden units and a *tanh* activation function, where the return_sequences parameter is set to *False*. The classification is performed via a final fully connected Dense layer with a Softmax activation function.

Table 6 represent all configuration of the four proposed deep learning models.

**Table 6:** Configuration of deep learning models.

Model	CNN layers	LSTM layer	Filters/hidden units	Activation function	Return sequences	Optimizer	Output layer
4 LSTM	-	4	256, 128, 64, 32	Tanh	True, True, True, False	Adam	Dense Softmax
2 CNN- 2LSTM	2	2	CNN: 512, 256 LSTM: 128, 128	Tanh	True, False	Adamx	Dense Softmax
3 CNN- 1 LSTM	3	1	CNN: 512, 256, 128 LSTM: 64	Tanh	False	Adamx	Dense Softmax
2 CNN-1 LSTM	2	1	CNN: 256, 256 LSTM: 128	Tanh	False	Adam	Dense Softmax

To evaluate the robustness and stability of the proposed architectures, each model was executed multiple times under the same experimental settings. Table 7 reports the average performance together with the standard deviation (STD) and the 95% confidence interval (CI). The relatively small standard deviation values indicate that the proposed models produce consistent results across repeated executions, while the narrow confidence intervals further confirm the reliability and statistical stability of the reported performance.

**Table 7:** Statistical comparison of the four evaluated architectures using macro-averaged performance metrics (mean ± standard deviation) and 95% confidence intervals.

Model	Macro precision	Macro recall	Macro F1-score	Confidence interval (95%)
4 LSTM	0.95 ± 0.0006	0.94 ± 0.0006	0.94 ± 0.0006	[0.945, 0.957]
2CNN-2LSTM	0.98 ± 0.0002	0.97 ± 0.0002	0.97 ± 0.0002	[. 9793,. 9796]
2 CNN-1 LSTM	0.97 ± 0.0004	0.94 ± 0.0006	0.95 ± 0.0004	[0.9506, 0.9516]
3CNN-1LSTM	0.94 ± 0.0007	0.92 ± 0.0017	0.92 ± 0.0015	[0.9188, 0.9242]

**Fig. 6:**
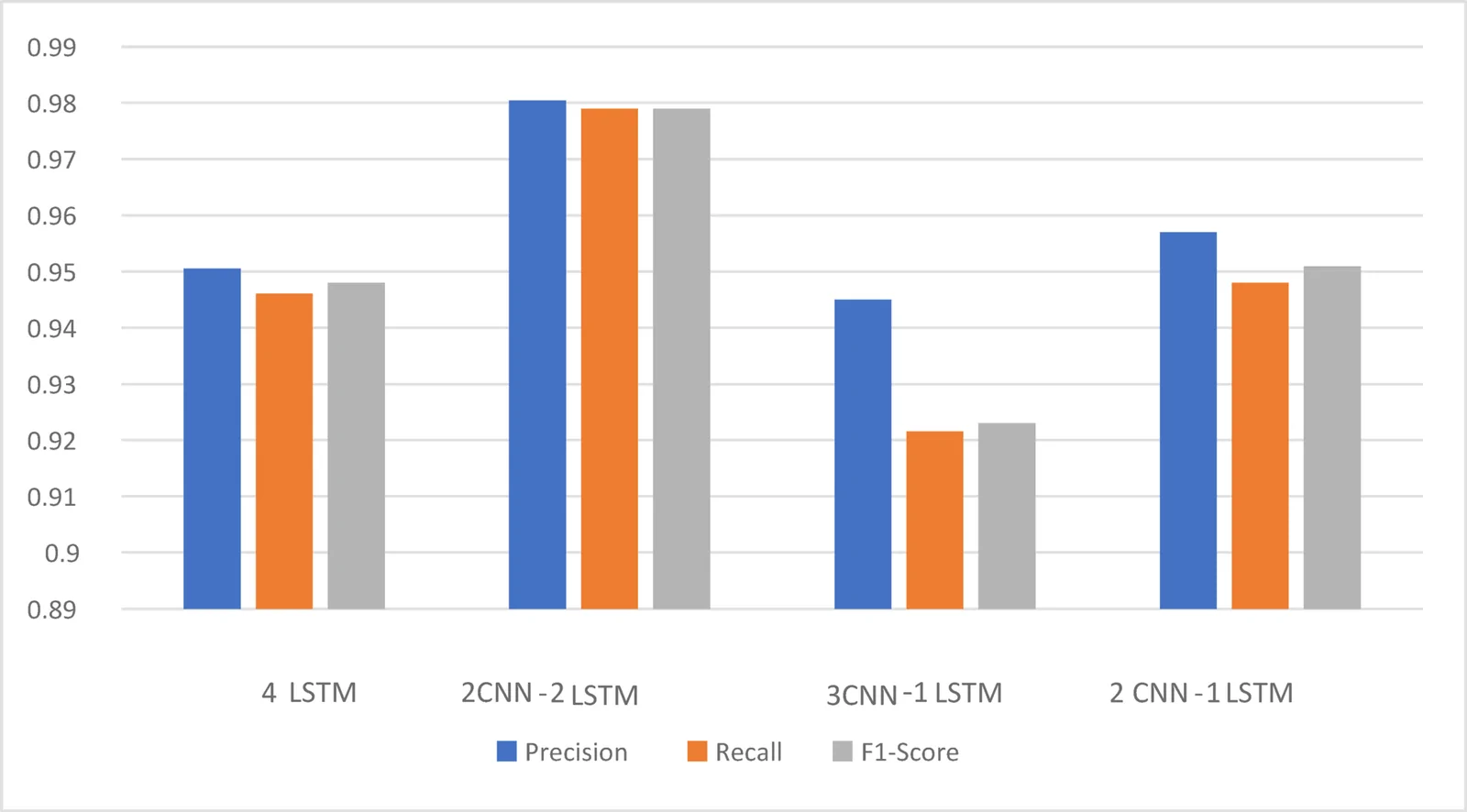
Performance comparison across four distinct architectural configurations.

All results of all different classes are represented in Table 8. A comparison between the 4 models is presented in Fig. 6.

**Table 8:** Results of all models.

Class	4 LSTM			2 CNN-2 LSTM			3 CNN-1LSTM			2 CNN-1LSTM		
	Precision	Recall	F1-score	Precision	Recall	F1-score	Precision	Recall	F1-score	Precision	Recall	F1-score
0	0.99	1	1	1	1	1	1	1	1	0.99	1	1
1	0.99	0.99	0.99	0.99	0.99	0.99	0.99	0.99	0.99	1	0.99	0.99
2	1	0.98	0.99	0.99	0.99	0.99	0.99	0.99	0.99	1	0.99	0.99
3	1	0.97	0.98	1	0.98	0.99	0.99	0.98	0.99	1	0.98	0.99
4	1	0.97	0.99	1	0.98	0.99	0.99	0.99	0.99	0.99	0.99	0.99
5	0.99	0.98	0.98	0.99	0.99	0.99	0.99	0.99	0.99	0.99	0.99	0.99
6	0.99	0.98	0.98	0.99	0.99	0.99	0.99	0.98	0.99	0.95	0.97	0.96
7	0.99	0.96	0.98	0.99	0.99	0.99	0.97	0.96	0.97	0.95	0.94	0.94
8	0.97	0.99	0.98	0.99	1	0.99	0.96	0.97	0.97	0.94	0.95	0.94
9	0.98	0.97	0.97	0.97	0.98	0.98	0.95	0.96	0.95	0.88	0.91	0.9
10	0.97	0.99	0.98	0.99	0.98	0.98	0.97	0.96	0.97	0.99	0.89	0.93
11	0.97	0.96	0.97	0.99	0.99	0.99	0.99	0.96	0.98	0.99	0.97	0.98
12	0.92	0.95	0.93	0.97	0.99	0.98	0.96	0.97	0.96	0.92	0.99	0.95
13	0.96	0.96	0.96	0.96	0.99	0.98	0.98	0.99	0.98	0.94	0.98	0.96
14	0.92	0.94	0.93	0.97	0.96	0.96	0.91	0.97	0.94	0.9	0.92	0.91
15	0.93	0.93	0.93	0.98	0.97	0.97	0.97	0.84	0.9	0.9	0.9	0.9
16	0.96	0.93	0.94	0.98	0.98	0.98	0.89	0.97	0.93	0.94	0.92	0.93
17	0.82	0.75	0.79	0.92	0.91	0.91	0.91	0.23	0.36	0.96	0.76	0.85
18	0.8	0.83	0.81	0.95	0.96	0.96	0.7	0.76	0.73	0.94	0.95	0.95
19	0.86	0.89	0.88	0.99	0.96	0.97	0.8	0.97	0.88	0.97	0.97	0.97
Macro _avg	0.95	0.95	0.95	0.98	0.98	0.98	0.94	0.92	0.92	0.96	0.95	0.95
Wighted_avg	0.97	0.97	0.97	0.99	0.99	0.99	0.97	0.97	0.96	0.97	0.97	0.97

**Fig. 7:**
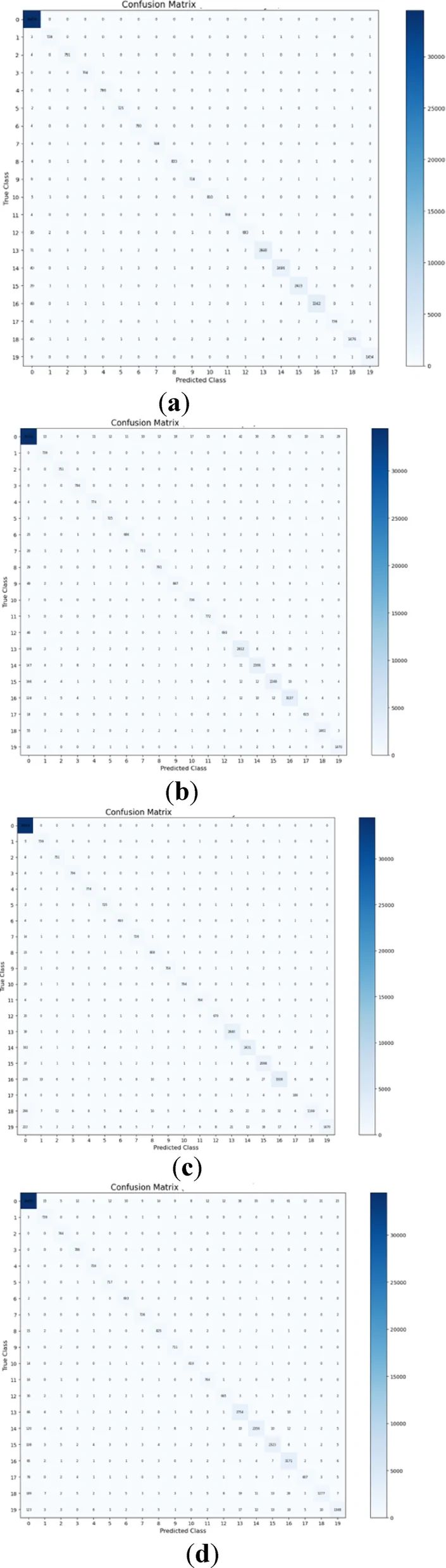
(**a**) 2-CNN-2LSTM, (**b**) 2 CNN-1 LSTM, (**c**) 3CNN-1LSTM, (**d**) 4 LSTM.

To further analyze the classification performance of the proposed models at a detailed level, the confusion matrix is presented. This allows for a clearer understanding of how well each class is correctly identified and highlights the types of misclassifications that occur between different attack categories. Figure 7 represents the confusion matrix for the four models.

**Fig. 8:**
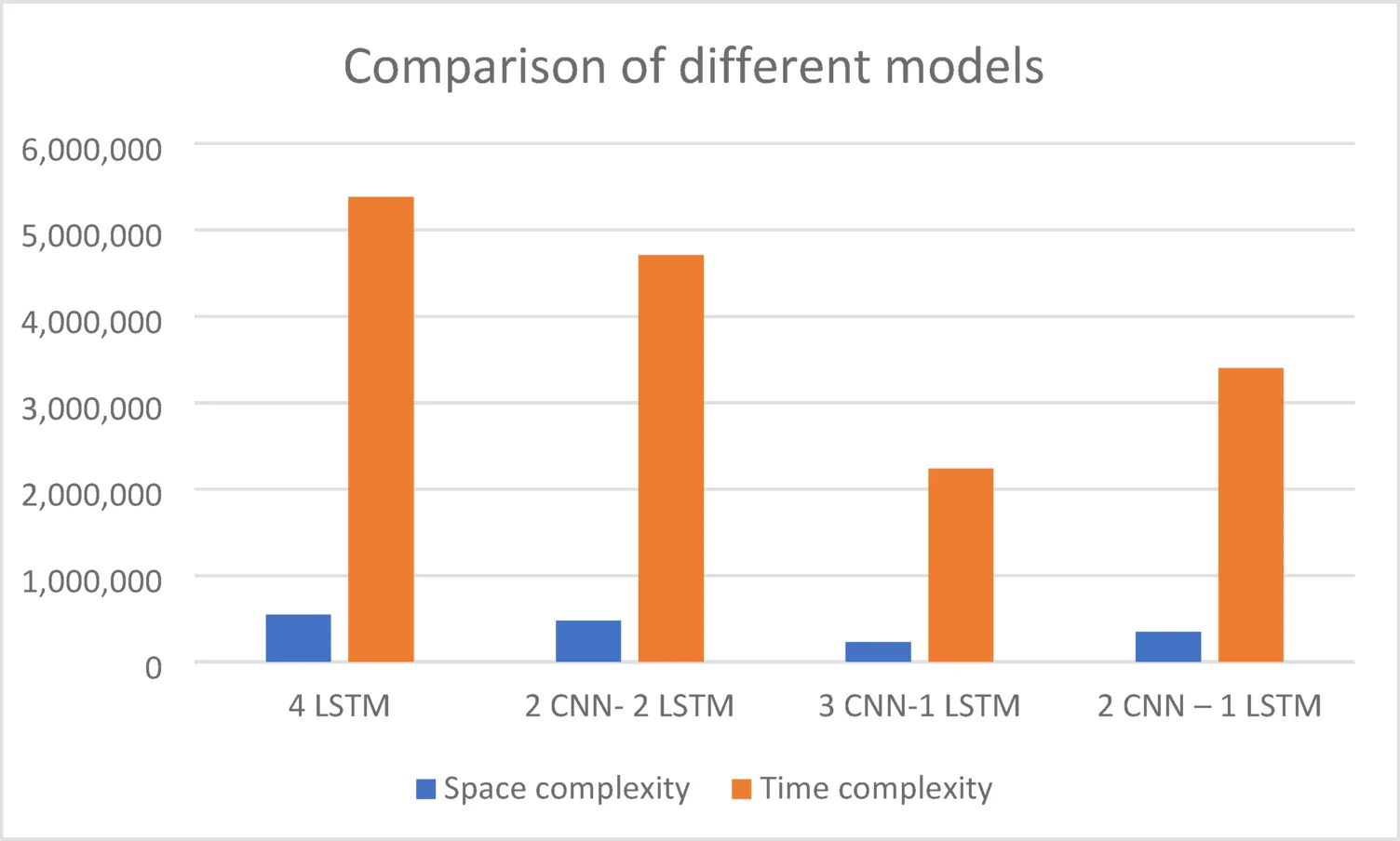
Comparison of computational space complexity and time across the evaluated deep learning models.

To provide a rigorous computational evaluation, the architectural complexity of the four proposed models was quantified using Floating-Point Operations (FLOPs) alongside empirical Execution Time (seconds per epoch for training, and milliseconds per sequence for testing). The theoretical FLOPs count was programmatically extracted using the Keras framework profiler, capturing the total mathematical operations across all convolutional kernels, recurrent gates, and fully connected layers. The results are represented in Table 9. Comparison between all models is represented in Fig. 8.

**Table 9:** Computational costs of all models.

Model	Space complexity (number of parameters)	Time complexity (FLOPs)	Training and testing time (seconds)
4 LSTM	545,076	5,383,744	5669
2 CNN- 2 LSTM	475,555	4,709,472	4269
3 CNN-1 LSTM	227,380	2,240,128	1934
2 CNN – 1 LSTM	349,268	3,398,912	2557

**Fig. 9:**
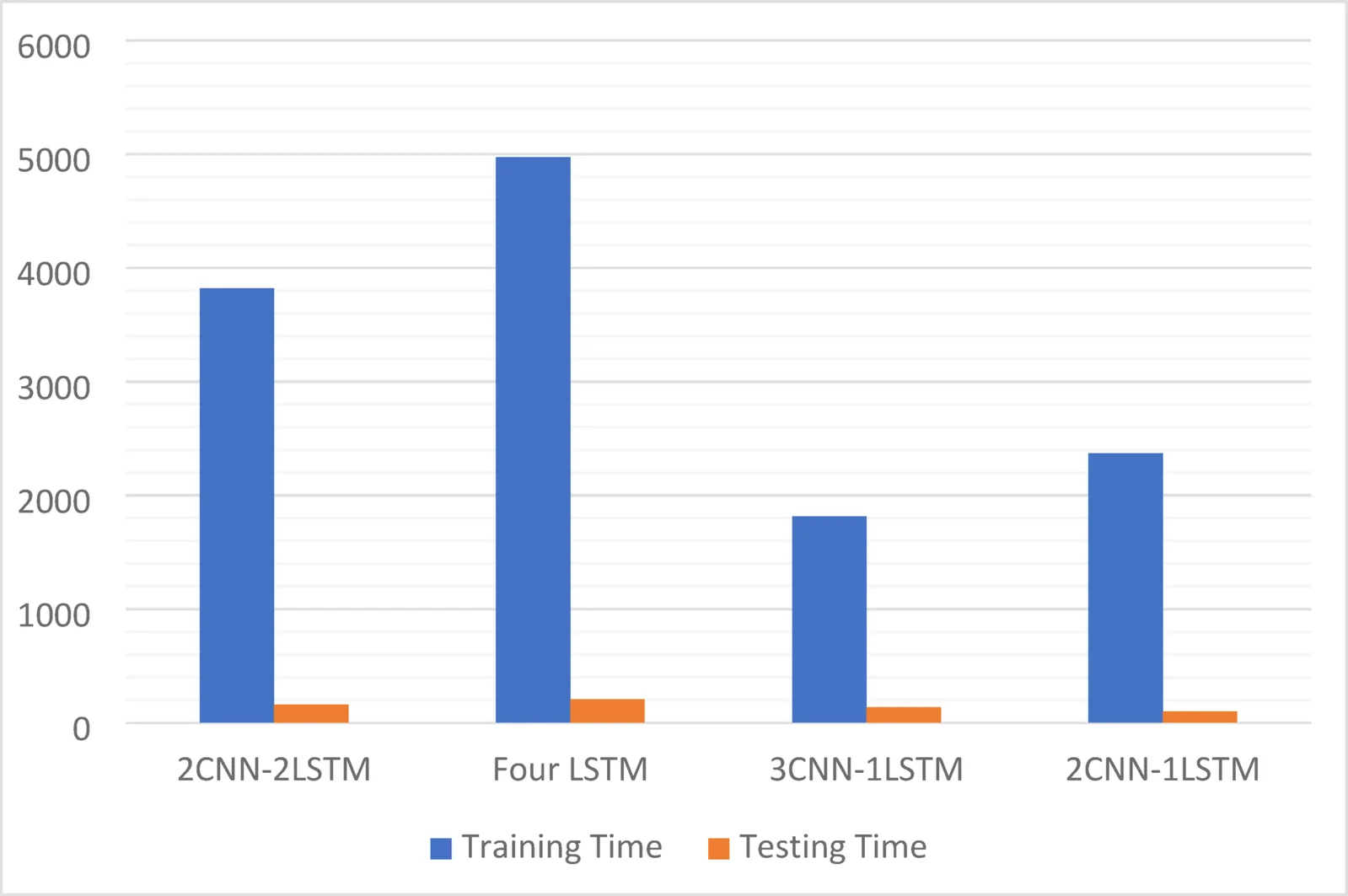
Comparison of training and testing time across the evaluated deep learning models.

The duration of training and testing is presented on Fig. 9.

Based on previous results, we can draw some conclusions:

i. As observed from the results, the proposed 2CNN-2LSTM model achieves the highest overall performance across all evaluation metrics, reaching a Macro Precision of * 0.98*, Macro Recall of * 0.97*, and Macro F1-Score of * 0.97*. Furthermore, the proposed architecture exhibits extremely low variability ($\:\pm\:\mathrm{SD}=0.0002$) and a very narrow $\:95\%$ Confidence Interval ($\:\left[\mathrm{0.9793,0.9796}\right]$) centered tightly around its mean F1-score. These results indicate that the model produces highly consistent and reproducible performance across repeated experimental runs, suggesting that its superior performance is stable rather than being an artifact of random parameter initialization.
ii. The 2 CNN–2 LSTM model achieved superior performance because of its efficient integration of spatial feature extraction (CNN) and temporal sequence modeling (LSTM), which is well-suited to the configuration of the VeReMi extension dataset. This dataset provides:
  - *Robust temporal relationships*: as the data is organized in sequences and is time-ordered for each vehicle.
  - *Spatial variability*: encompassing features such as position, velocity, direction, and noise that differ among vehicles and situations.
  - *Hierarchical patterns*: where minor anomalies (such as changes in speed) combine to form more significant misbehavior.
iii. According to the specifications of the dataset, the 2CNN-2LSTM achieved the highest scores as it is made up of:
  - The initial CNN layer detects basic spatial features, such as abrupt changes in speed or direction.
  - The subsequent CNN layer extracts more complex indicators of misbehavior across different input dimensions.
  - The first LSTM layer captures short-term temporal variations.
  - The second LSTM layer analyzes longer-term behavioral patterns, helping the model differentiate between temporary fluctuations and persistent attack behaviors.
iv. Additionally, the implementation of the Adamax optimizer, known for its effectiveness with sparse gradients and noisy features (often present in VANET data), facilitated a more stable and quicker convergence.
v. While this architecture is computationally heavier than others, the synergy between CNN and LSTM layers allows it to generalize better across different types of misbehavior.
vi. A closer look at how each class performs shows that not all classes are equally easy to learn. Class 17 consistently has the lowest precision, recall, and F1-score across all models. This drop in performance mainly comes from the very small number of training sequences for this class. This limitation makes it hard for the model to recognize its unique patterns.
vii. Additionally, some attack classes have similar spatial and temporal characteristics, which makes it hard to tell them apart. Since VANET data includes noisy mobility patterns and very changing behaviors, some misbehaviors may show up with similar feature distributions. This leads to confusion between classes.
viii. In contrast, architectures with less balanced layer combinations (such as the 3CNN-1LSTM model) demonstrated both lower overall performance ($\:\mathrm{F1-score}=0.92$) and higher performance variance ($\:\pm\:\mathrm{SD}=0.0015$). This empirical evidence confirms that integrating a balanced feature extraction stack (2 CNN layers for spatial feature representation combined with 2 LSTM layers for temporal sequence learning) provides the optimal architectural stability required for misbehavior detection in VANETs.
ix. The previous conclusion is assured by model 2CNN-1LSTM. The precision, recall, and F1 scores are 95.7%, 94.8%, and 95.1%, respectively. However, it took second place in training and testing time.
x. 4 LSTM performed better in contrast to 3CNN-1LSTM. It has average precision, recall, and F1-score values of 95.05%, 94.6%, and 94.8%, respectively. On the other hand, it took the most time for training and testing.
xi. The 4 LSTM model exhibits the highest values regarding training and testing duration, as well as space and time complexity.

The results could be applied in real-world situations across various dimensions. Firstly, they may aid in the early detection of accidents, facilitate communication between vehicles, and support predictive safety mechanisms. By assessing vehicle behavior in real-time, our classifiers can pinpoint potential collision trends. This enhances road safety and decreases the likelihood of accidents. Additionally, the various models allow fog nodes to make well-informed decisions based on historical and current data. This contributes to adaptive traffic management and improved path planning.

To ensure a robust evaluation, each model was trained and tested over five independent runs. The results are reported for Precision, Recall, and F1-score.

**Table 10:** Results of the model in the second classifier.

Feature Scenario	Accuracy	Macro_Precision	Macro_recall	Macro_F1-Score	Confidence _interval 95%
Scenario 1: baseline configuration	0.98±0.0004	0.97±0.0004	0.96±0.0004	0.96±0.0003	[0.965,0.966]
Scenario 2: speed & position only	0.95±0.0012	0.90±0.0004	0.88±0.0021	0.88±0.0019	[0.959,0.960]
Scenario 3: minimal feature subset	0.80±0.0035	0.46±0.0035	0.45±0.0033	0.45±0.0033	[0.459,0.470]
Scenario 4: with all features (proposed)	0.99±0.0002	0.98±0.0002	0.98±0.0002	0.98±0.0002	[0.978, 0.982]

To evaluate the statistical significance and feature dependence of our best-performing model (2-CNN-2-LSTM), an extensive ablation study was conducted across 5 randomized runs under four distinct feature environments (Table 10).

The results shown demonstrate that the model is highly stable yet sensitive to data richness. Under Scenario 3 (minimal features), the macro F1-score sharply drops to 0.45, exposing the insufficiency of relying on restricted inputs. Incorporating spatial-temporal dynamics via speed and position metrics (Scenario 2) elevates the detection accuracy to 95%. However, the optimal performance is unlocked in Scenario 4 (With All Features), yielding a stable peak accuracy of 99% and a macro F1-score of 0.98. The logical progression of these results across repeated trials mathematically confirms that the 2-CNN-2-LSTM model is capturing authentic structural mobility patterns rather than capitalizing on single-run initialization anomalies.

### Second classifier

**Fig. 10:**
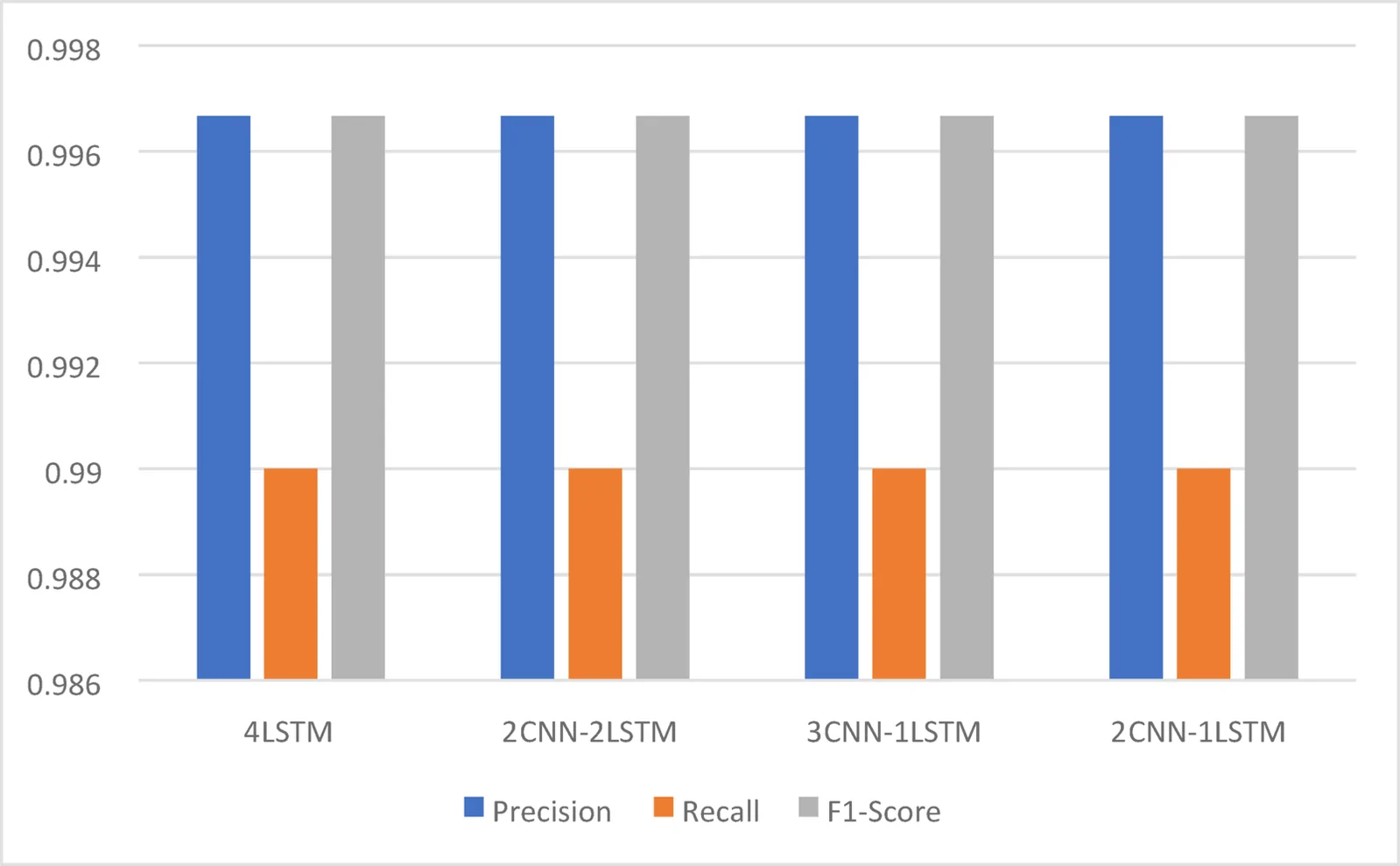
Performance comparison (Precision, Recall, and F1-Score) across four evaluated proposed model architectures.

**Fig. 11:**
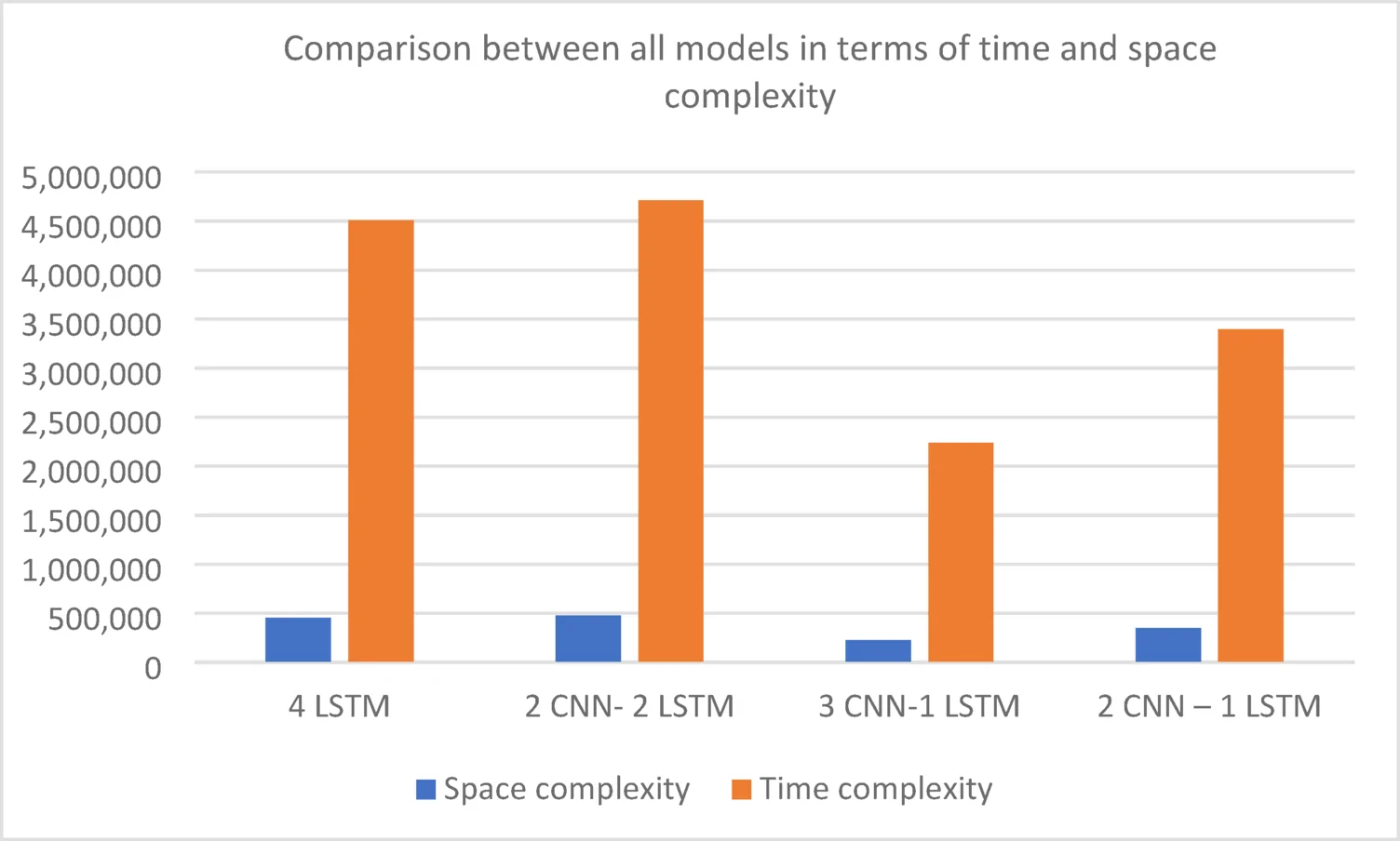
Comparison of computational space complexity and time across the evaluated deep learning models.

**Fig. 12:**
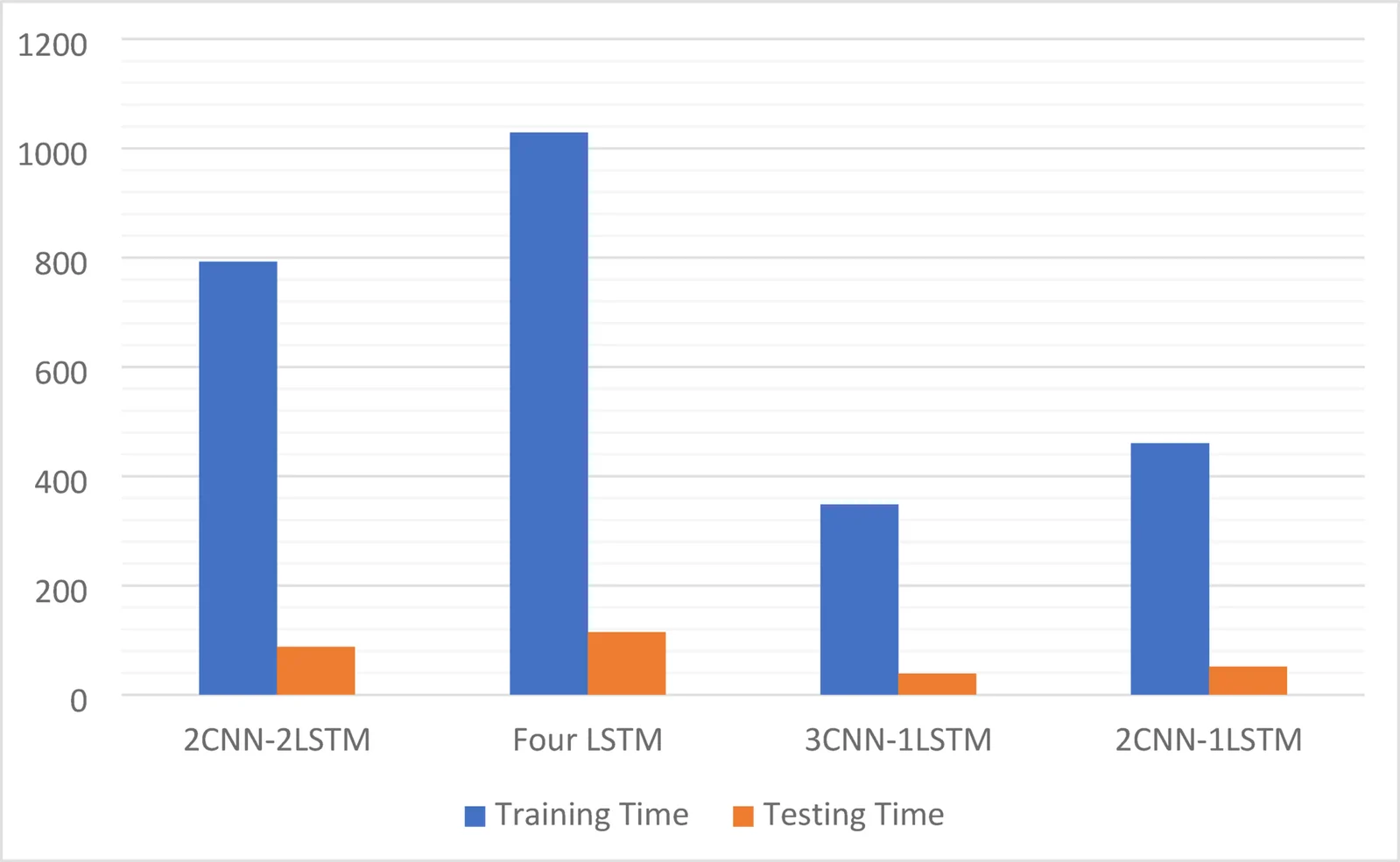
Comparison of training and testing time across the evaluated deep learning models.

The same models in the first classifier were used with the same parameters. The results of all models are mentioned in Table 11. Figure 10 shows a chart comparing the results of four models. Computational costs in terms of space complexity and time complexity are represented in Table 12. Comparison between all models is represented in Fig. 11. Finally, the Time complexity for all models is represented in Fig. 12.

**Table 11:** Results of the model in the second classifier.

Class	4LSTM			2CNN-2LSTM			3 CNN-1LSTM			2CNN-1LSTM		
	Precision	Recall	F1-score	Precision	Recall	F1-score	Precision	Recall	F1-score	Precision	Recall	F1-score
0	1	1	1	0.99	1	1	0.99	1	1	1	1	1
1	1	0.99	0.99	1	0.98	0.99	0.99	0.99	0.99	0.99	0.99	0.99
2	1	1	1	1	0.99	1	1	0.99	0.99	1	1	1
Macro_ avg	0.99	0.99	0.99	0.99	0.99	0.99	0.99	0.99	0.99	0.99	0.99	0.99
Weighted_avg	0.99	0.99	0.99	0.99	0.99	0.99	0.99	0.99	0.99	0.99	0.99	0.99

As shown, all models have the best results in all categories.

**Table 12:** Computational costs of all models.

Model	Space complexity (number of parameters)	Time complexity (FLOPs)	Training and testing time (seconds)
4 LSTM	452,171	4,509,280	1143
2 CNN- 2 LSTM	476,116	4,710,016	880
3 CNN-1 LSTM	226,819	2,239,584	386
2 CNN-1 LSTM	348,163	3,397,824	511

Figure 11 shows that the second classifier requires approximately 20% of the execution time of the first classifier. This reduction in computational overhead makes the second classifier more suitable for resource-constrained fog nodes and real-time decision-making scenarios, where rapid detection is often more critical than detailed attack attribution.

## Discussion

To provide an enhanced comparison with other work, the overall average metric was computed according to Eq. (5):

5 $$\:{M}_{avg=}{\sum\:}_{0}^{n}{m}_{n}$$

Where m is any of the metrics, precision, recall, and f1-score for the nth class (from 0 to 19 in the first classifier and from 0 to 2 for the second classifier).

**Fig. 13:**
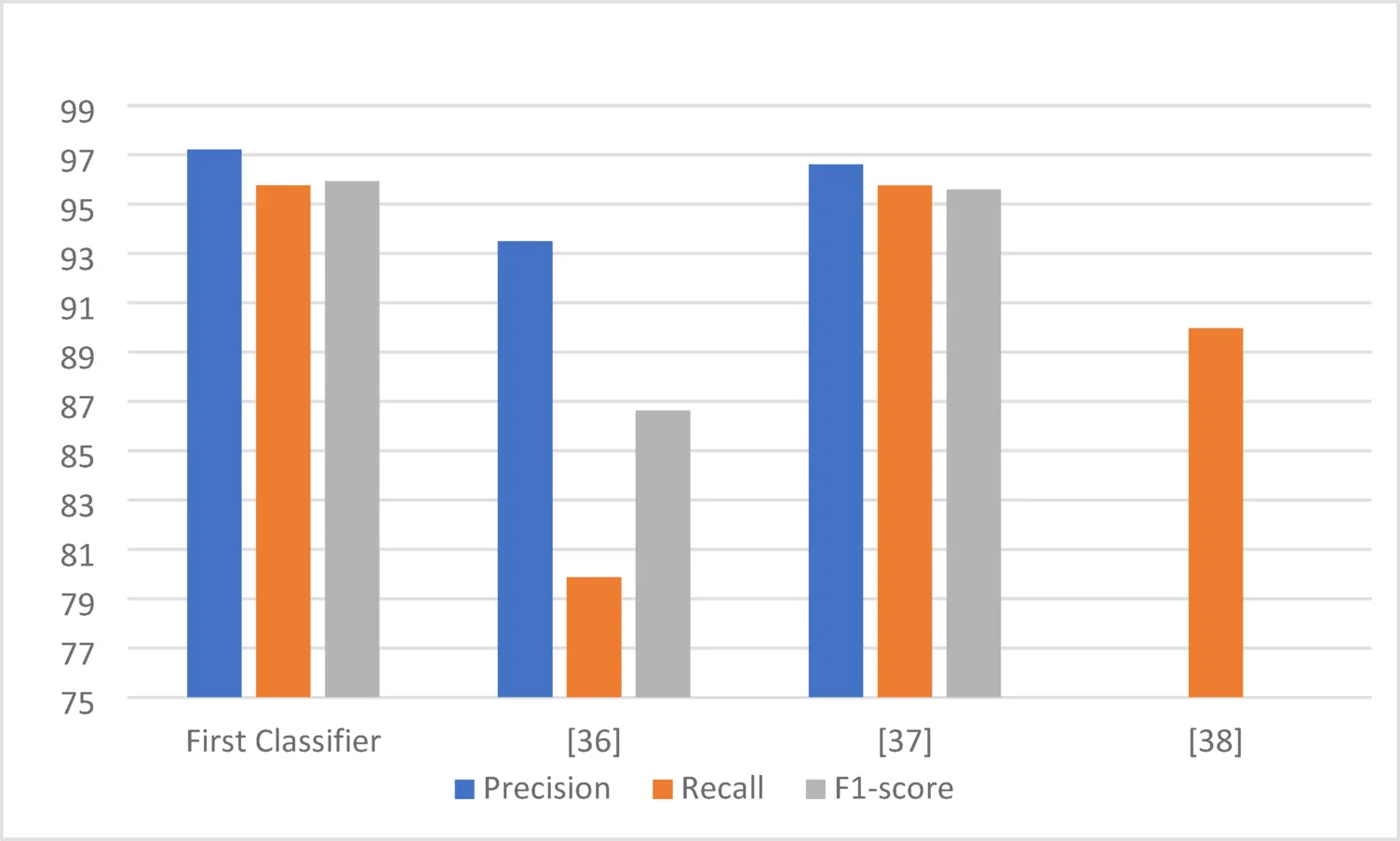
Performance comparison of the proposed model (First Classifier) against existing papers^41–43^.

Figure 13 provides a performance comparison of the proposed work with the existing studies on^41–43^. The proposed work outperforms the other work in the precision metric. It can also be noticed that our work barely differentiates from^42^ in recall and F1-score. Otherwise, it shows the best results in comparison with^41,43^.

**Fig. 14:**
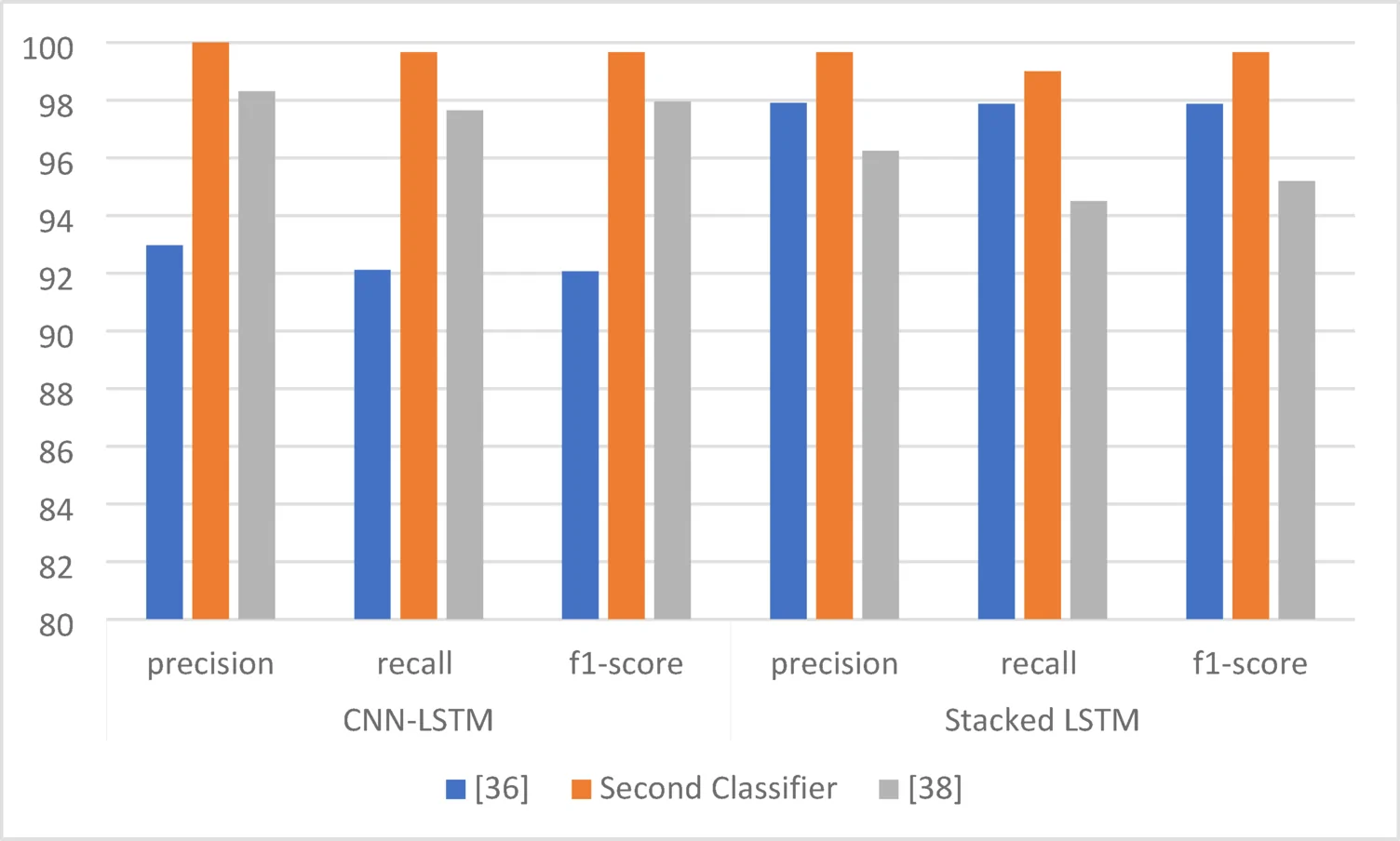
Comparative evaluation of the second classifier (CNN-LSTM vs. Stacked LSTM) against existing benchmark studies^41,43^.

In the second classifier, it is preferred to compare with the work that classifies the category of vehicles. As shown on Fig. 14, our work outperforms other work either in the case of using a stacked LSTM model or a CNN-LSTM model.

Despite the encouraging results from various models, there are numerous challenges that these models may encounter. In scenarios with high levels of noise, our model encounters difficulties when handling noisy, incomplete, or unstructured data. This issue may arise from malfunctions in the OBU, resulting in reduced performance of the classifiers. To tackle this challenge, techniques such as data augmentation or denoising autoencoders can be implemented. In a dynamic environment, the temporal patterns that the models learn may change rapidly. Consequently, the LSTM layers must adapt quickly to these swift changes. To overcome this challenge, incorporating reinforcement learning techniques could be beneficial. This approach enables the model to continuously modify its parameters in response to new data.

## Conclusion

VANETs are a hot topic of research nowadays. Building a reliable and safe system is a challenging point. Integrating fog computing with VANETs becomes necessary for real-time applications. The old techniques for securing VANETs have become insufficient. For that, the emergence of an intrusion detection system has become necessary due to the importance of safety messages.

In this study, the aim was to create a system that aids in the distribution of safety messages. Additionally, making sure the car was not broadcasting data from an attacker or just had a system mistake. Based on the VeReMi extension dataset, a deep learning-based intrusion detection system was proposed that can be implemented on fog servers. Two different classifiers were suggested: one for all categories and the other specifically for the vehicle category. The results that appeared from the experiment showed that the models used the first classifier, which scored from 94.5% to 97% according to the F1 score. The models used in the second classifier scored a performance that reached 99.7% for the F1 score. The outcomes of the four alternative models were compared with those of the other work.

Despite the promising results, several limitations should be acknowledged. First, the proposed framework was evaluated using only the VeReMi extension dataset, which may not fully represent all real-world VANET environments. Second, external validation using additional VANET datasets was not conducted, limiting the assessment of the model’s generalization capability across different traffic scenarios and attack patterns. Third, Network simulation environments such as SUMO, NS-3, or OMNeT + + were not employed, as the focus of this work is on classification performance rather than network-level system simulation. Extending the proposed framework to a full network simulation environment remains an important direction for future work. Finally, the study focused primarily on detection performance and computational characteristics, while communication-level aspects such as end-to-end network latency and large-scale deployment constraints were not investigated.

Despite these limitations, the proposed framework demonstrates promising potential for deployment in VANET misbehavior detection scenarios. The use of general-purpose machine learning and deep learning models, combined with message-level behavioral analysis, enables the framework to be adapted to different vehicular communication environments. However, further validation on heterogeneous datasets and real-world experimental platforms is required to confirm its generalization capability.

Based on our results, future work can concentrate on increasing the number of messages of type 17. While this study established core classification efficiency, the proposed models will be evaluated in realistic, large-scale fog computing deployment scenarios. This includes conducting full network-level simulations (using tools such as SUMO coupled with NS-3 or OMNeT++) to explicitly analyze practical constraints, such as RSU processing capacity, real-time edge-server throughput, memory consumption, computational overhead, edge-server throughput, end-to-end network latency, and physical channel congestion under high vehicle densities. These future investigations will provide a deeper empirical understanding of the scalability and real-world applicability of the proposed intrusion detection framework in dense urban VANET environments.

## Data Availability

The dataset used in this study is openly available online. The dataset can be accessed through the following public repositories: https://github.com/josephkamel/VeReMi-Dataset-https://mega.nz/folder/z0pnGA4a#WFEUISyS5_maabhcEI7HQA/folder/r8ATxa7D.
